# More efficient North Atlantic carbon pump during the Last Glacial Maximum

**DOI:** 10.1038/s41467-019-10028-z

**Published:** 2019-05-15

**Authors:** J. Yu, L. Menviel, Z. D. Jin, D. J. R. Thornalley, G. L. Foster, E. J. Rohling, I. N. McCave, J. F. McManus, Y. Dai, H. Ren, F. He, F. Zhang, P. J. Chen, A. P. Roberts

**Affiliations:** 1 Research School of Earth Sciences, The Australian National University, Canberra, ACT 2601 Australia; 2 SKLLQG, Institute of Earth Environment, Chinese Academy of Sciences, Xi’an, 710061 China; 3 Climate Change Research Centre, University of New South Wales, Sydney, NSW 2052 Australia; 4 Open Studio for Oceanic-Continental Climate and Environment Changes, Qingdao National Laboratory for Marine Science and Technology, Qingdao, 266061 China; 5 Department of Geography, University College London, London, WC1E 6BT UK; 6 Ocean and Earth Science, University of Southampton, National Oceanography Centre, Southampton, SO14 3ZH UK; 7 Department of Earth Sciences, University of Cambridge, Cambridge, CB2 3EQ UK; 8 Lamont-Doherty Earth Observatory of Columbia University, 61 Route 9W/PO Box 1000, Palisades, NY 10964-8000 USA; 9 Department of Geosciences, National Taiwan University, Taipei, Taiwan; 10 Center for Climatic Research, Nelson Institute for Environmental Studies, University of Wisconsin-Madison, Madison, WI 53706 USA; 11 College of Earth, Ocean, and Atmospheric Sciences, Oregon State University, Corvallis, OR 97331 USA; 12 CAS Center for Excellence in Quaternary Science and Global Change, Xi’an, 710061 China; 13 State Key Laboratory of Marine Geology, Tongji University, Shanghai, 200092 China

**Keywords:** Carbon cycle, Biogeochemistry, Palaeoceanography, Palaeoclimate

## Abstract

During the Last Glacial Maximum (LGM; ~20,000 years ago), the global ocean sequestered a large amount of carbon lost from the atmosphere and terrestrial biosphere. Suppressed CO_2_ outgassing from the Southern Ocean is the prevailing explanation for this carbon sequestration. By contrast, the North Atlantic Ocean—a major conduit for atmospheric CO_2_ transport to the ocean interior via the overturning circulation—has received much less attention. Here we demonstrate that North Atlantic carbon pump efficiency during the LGM was almost doubled relative to the Holocene. This is based on a novel proxy approach to estimate air–sea CO_2_ exchange signals using combined carbonate ion and nutrient reconstructions for multiple sediment cores from the North Atlantic. Our data indicate that in tandem with Southern Ocean processes, enhanced North Atlantic CO_2_ absorption contributed to lowering ice-age atmospheric CO_2_.

## Abstract (web summary)

Atmospheric CO_2_ is governed by CO_2_ gains (e.g., via Southern Ocean outgassing) and losses (e.g., via North Atlantic absorption). Using a novel method to estimate air–sea CO_2_ exchange signals, the authors show that North Atlantic CO_2_ absorption became more efficient and contributed to lowering atmospheric CO_2_ during ice ages.

## Introduction

The North Atlantic Ocean (>~35°N, including the Nordic Seas and Arctic Ocean) is a major atmospheric CO_2_ sink, which has been mitigating anthropogenic atmospheric CO_2_ increases^1^. Preindustrial North Atlantic surface water partial pressure of CO_2_ (*p*CO_2_) was up to ~100 μatm lower than the contemporary atmospheric *p*CO_2_ of ~280 μatm, which caused substantial atmospheric CO_2_ invasion^2,3^. Despite its modest area, the North Atlantic Ocean accounts for at least ~30% of the global ocean CO_2_ uptake today and during preindustrial times^1,4^. Over longer timescales, large-scale oceanic carbon sequestration also occurred during Plio-Pleistocene glaciations^5–7^. This is commonly attributed to reduced glacial Southern Ocean CO_2_ outgassing^6,8,9^, while even the sign of past North Atlantic CO_2_ uptake efficiency changes remains unconstrained. Here, we present a novel proxy approach to trace atmospheric CO_2_ invasion in the North Atlantic and thereby evaluate its role in carbon sequestration in ice-age oceans. We find that the last glacial North Atlantic carbon absorption became more efficient, highlighting a critical role of the North Atlantic Ocean in regulating glacial–interglacial atmospheric CO_2_ changes.

## Results

### Air–sea CO_2_ exchange tracers

**Fig. 1:**
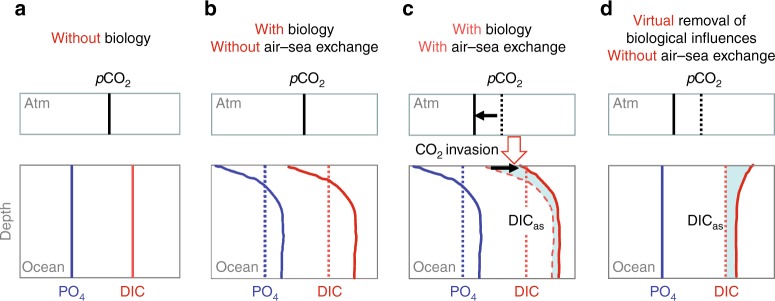
Concepts to distinguish DIC_as_. For simplicity, only CO_2_ invasion associated with organic matter cycling is considered. In the ocean box, vertical solid and dashed lines (**a**–**d**) represent mean PO_4_ (blue) and DIC (red) in an abiotic ocean (**a**). Biology redistributes DIC and PO_4_ following Redfield stoichiometry (curves; **b**). This decreases surface-ocean DIC and *p*CO_2_, and hence causes air-to-sea CO_2_ transfer (**c**). Through mixing and ocean circulation, CO_2_ invasion raises water-column DIC, i.e., shifting dashed curve (equals the red-solid curve in **b**) to red-solid curve (**c**). The shaded region in **c** represents air–sea exchange DIC_as_ signatures. After removing carbon redistribution by biology based on PO_4_-related curvature of the profiles (**b**), DIC_as_ can be revealed by the shaded region in **d**

Any effect of ocean processes on atmospheric *p*CO_2_ must occur via air–sea CO_2_ exchange. In the North Atlantic, high-nutrient utilization decreases surface-water dissolved inorganic carbon (DIC) and causes surface-water *p*CO_2_ to be lower than atmospheric *p*CO_2_ (Supplementary Fig. 1). This leads to net air-to-sea CO_2_ transfer, creating an air–sea exchange signature of DIC (DIC_as_). DIC_as_ signals can be distinguished by accounting for within-ocean DIC redistributions that are heavily mediated by biology (Fig. 1). Biological cycling of organic matter depletes DIC and nutrients such as phosphate (PO_4_) in surface waters and enriches them at depth. Seawater mixing also affects DIC and PO_4_ concentrations in the ocean. Nevertheless, PO_4_ variations are ultimately determined by biological processes: without biology, PO_4_ should be the same everywhere in the ocean regardless of ocean circulation (ignoring the small effect from salinity change). Because marine biology incorporates and releases PO_4_ and DIC in a relatively fixed proportion following Redfield stoichiometry^3,10^ and because PO_4_ is not affected by air–sea exchange, PO_4_ can be used to estimate biology-driven within-ocean DIC redistributions (Fig. 1). Any within-ocean DIC redistribution associated with CaCO_3_ cycling can be accounted for using alkalinity (ALK) and nitrate.

**Fig. 2:**
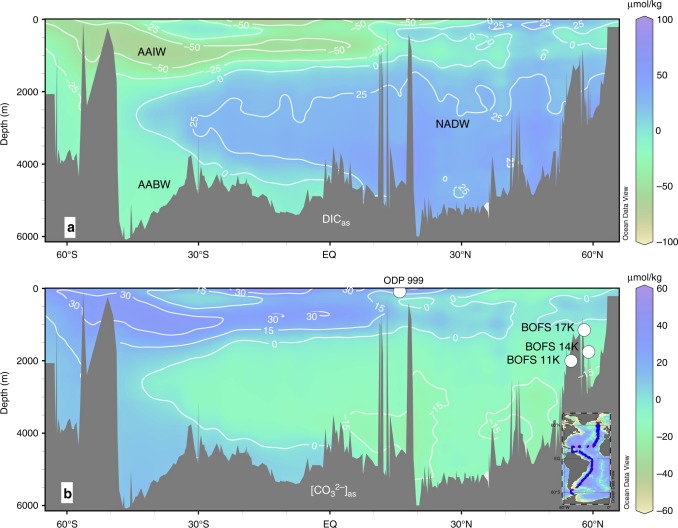
Preindustrial Atlantic air–sea exchange tracers. **a** DIC_as_. **b** [CO_3_^2−^]_as_. Circles represent studied sediment cores. Inset: GLODAP hydrographic data^2^ used to generate the sections^96^. NADW North Atlantic deep water, AABW Antarctic bottom water, AAIW Antarctic intermediate water. See Methods for calculation details

Following the established method^3^ to account for within-ocean DIC redistributions by soft-tissue and CaCO_3_ cycling, we calculate preindustrial Atlantic DIC_as_ using the GLODAP dataset^2^ (Fig. 2a). See Methods for details to calculate DIC_as_. More positive DIC_as_ values indicate a greater degree of atmospheric CO_2_ invasion. At basin-scale, the preindustrial DIC_as_ of North Atlantic deep water (NADW) is ~50–80 µmol/kg higher than for Antarctic bottom water (AABW) and Antarctic intermediate water (AAIW). This difference reflects North Atlantic CO_2_ uptake and Southern Ocean release^3,11^. North Atlantic CO_2_ absorption is driven by (i) an efficient solubility pump due to strong cooling of northward-flowing Gulf Stream waters and (ii) a strong biological pump associated with high nutrient utilization^12–14^. NADW thus represents an efficient pathway for atmospheric CO_2_ sequestration^6,15^. Through global deep ocean circulation, CO_2_ absorbed in the North Atlantic is transported throughout the world ocean^1,3^, with profound implications for the global carbon cycle.

No proxy exists to reconstruct past seawater DIC and ALK at acceptable precision for direct application, so we employ a linked carbonate system parameter for palaeoceanographic studies. Everything else being equal, atmospheric CO_2_ invasion would decrease seawater carbonate ion concentration ([CO_3_^2^^−^]), because CO_2_ reacts with carbonate ion to form bicarbonate^16^. We thus develop a new tracer, [CO_3_^2−^]_as_, which essentially reflects seawater [CO_3_^2^^−^] contrasts for the same biological (i.e., PO_4_) and physical (i.e., temperature–salinity–pressure; T–S–P) conditions (Fig. 2b; see Methods for calculation details). To extract air–sea exchange signals, it is necessary to compare [CO_3_^2−^] at the same PO_4_–T–S–P conditions because we must first remove influences on [CO_3_^2−^] from (i) within-ocean DIC and ALK redistributions by biology and (ii) T−S–P variations via their effects on CO_2_ system dissociation constants^16^. In the preindustrial Atlantic, the strong negative correlation between [CO_3_^2^^−^]_as_ and DIC_as_ (Fig. 2, Supplementary Fig. 2) indicates that [CO_3_^2−^]_as_ variations are affected only by DIC_as_, and thus are ultimately linked to air–sea CO_2_ exchange.

The Gulf Stream is a major NADW source^17^; thus, comparing the [CO_3_^2−^]_as_ gradient between the Gulf Stream and NADW can provide a measure of CO_2_ sequestration intensity during transformation of Gulf Stream waters into NADW. Because Gulf Stream waters are more or less in equilibrium with atmospheric *p*CO_2_ from ~10°N to 35°N^1,2^, the Gulf Stream−NADW [CO_3_^2−^]_as_ gradient mainly reflects North Atlantic (>~35°N) air–sea CO_2_ exchange (Supplementary Fig. 3). Physical oceanographers have shown that the path of Gulf Stream waters, rather than being a direct conveyor to the polar North Atlantic, is instead a “corkscrew”, where Gulf Stream waters are recirculated south in the subtropical gyre and subduct after being made more dense by air–sea heat loss (e.g., refs. ^18,19^). However, our interest lies in net CO_2_ uptake by the North Atlantic region, and variations in spatial pathways from Gulf Stream to NADW formation sites^18,19^ should not significantly complicate our conclusion. The greater the [CO_3_^2−^]_as_ gradient between Gulf Stream and NADW (instead of their absolute [CO_3_^2^^−^]_as_ values), the more efficient air–sea CO_2_ absorption by the North Atlantic. Linked to large-scale overturning circulation, Gulf Stream−NADW [CO_3_^2−^]_as_ gradient changes regulate long-term CO_2_ sequestration into the deep ocean.

### Downcore reconstructions

**Fig. 3:**
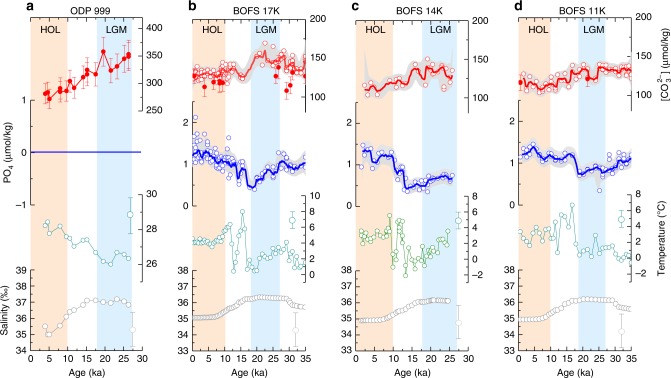
Down core reconstructions. **a** ODP 999. **b** BOFS 17 K. **c** BOFS 14 K. **d** BOFS 11 K. Seawater [CO_3_^2−^] values are derived from benthic B/Ca (empty circles) and δ^11^B (solid circles). Light gray envelopes and error bars: 2*σ*. Note different *y*-scales for surface- (ODP 999) and deep-water (BOFS cores) reconstructions. See Methods for reconstruction details

Next, we reconstruct past Gulf Stream–NADW [CO_3_^2−^]_as_ gradients to investigate North Atlantic carbon pump efficiency during the LGM (18–27 ka). Previous work suggests that most of North Atlantic subtropical gyre water circulates through the Caribbean Sea before being transported to the subpolar North Atlantic via the Gulf Stream^20^. We, therefore, use Caribbean Sea ODP Site 999 (12.8°N, 78.7°W) to constrain past Gulf Stream physicochemical conditions (Fig. 3, Supplementary Figs. 4 and 5). The feasibility of using ODP Site 999 to reflect the first-order Gulf Stream carbonate chemistry changes between the Holocene and LGM is supported by observations that (i) Caribbean surface waters have similar [CO_3_^2−^]_as_ values to hydrographic sites located within Gulf Stream during the preindustrial (Supplementary Fig. 3), and (ii) cores from the broader western subtropical Atlantic show comparable Holocene and LGM [CO_3_^2^^−^]_as_ signatures as those from ODP 999 (Supplementary Fig. 6). Surface-water T and S are estimated from *Globigerinoides ruber* Mg/Ca and sea level fluctuations, respectively^21,22^. Previously published *G. ruber* δ^11^B (ref. ^21^) is used to calculate surface-water pH, while ALK is estimated from S using the modern relationship between S and ALK^21,22^. Along with T, S, and ALK estimates, pH is then used to calculate surface-water [CO_3_^2^^−^] and DIC. Given the constraint from pH, seawater ALK and DIC must vary systematically within the ocean carbonate system (Supplementary Fig. 5). This allows precise estimation of [CO_3_^2−^], because even large ALK uncertainties (100 μmol/kg; ± 2*σ*, used throughout) only have a minor effect on [CO_3_^2−^] (~14 μmol/kg). Given its oligotrophic setting, past surface-water PO_4_ at ODP 999 is assumed to be zero^2,21,22^.

Three cores are used to reconstruct deep-water conditions of northern-sourced waters (Fig. 3). BOFS 17 K (58°N, 16.5°W, 1150 m) and BOFS 14 K (58.6°N, 19.4°W, 1756 m) are located close to the previously surmised center of Glacial North Atlantic intermediate water (GNAIW)^23^, while BOFS 11 K (55.2°N, 20.4°W, 2004 m) is thought to be affected by glacial Nordic Sea overflows^24^. We employ benthic foraminiferal δ^11^B and B/Ca to reconstruct deep-water [CO_3_^2−^] with an uncertainty of ~10 μmol/kg^25^. δ^11^B and B/Ca give consistent downcore [CO_3_^2−^] reconstructions. Benthic Cd/Ca is used to estimate deep-water Cd and PO_4_ based on an established approach (Supplementary Fig. 7)^26,27^. Past deep-water T and S changes are estimated from foraminiferal δ^18^O and sea level fluctuations; use of other methods negligibly affects our conclusion. In total, we present 180 new measurements for benthic foraminiferal δ^11^B, B/Ca, and Cd/Ca. Details of core materials, methods, new and compiled data, and fully propagated uncertainties are given in Methods and Supplementary Data 1–9.

### A pragmatic recipe to estimate [CO_3_^2−^]_as_ change

**Fig. 4:**
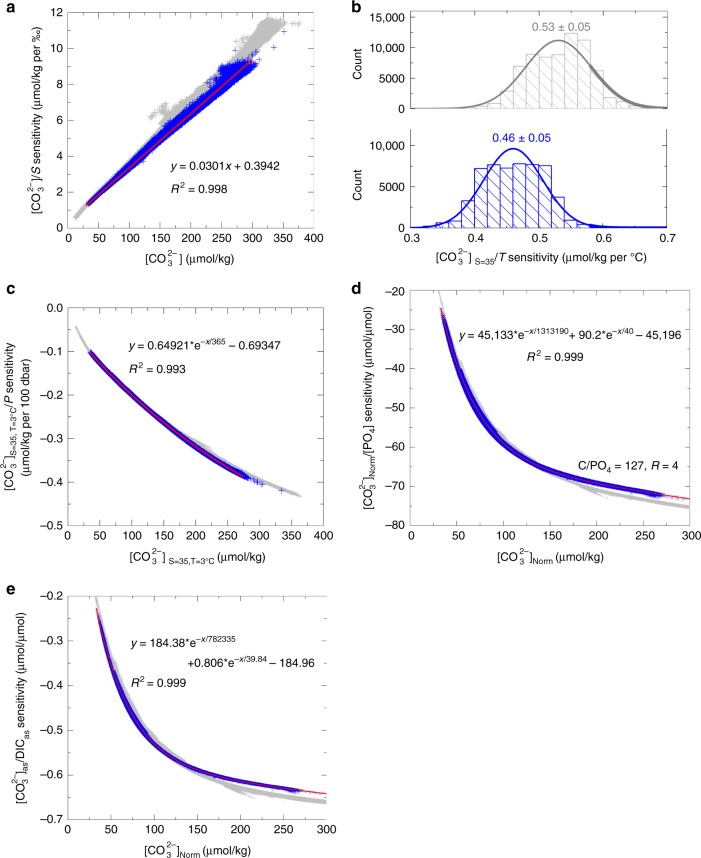
Carbonate system sensitivities to various changes. **a** Salinity effect. **b** Temperature effect. **c** Pressure effect. **d** Biological effect. **e** Air–sea CO_2_ exchange effect. Calculations are based on GLODAP^2^ (*n* = 55,399; blue) and a LGM output from LOVECLIM^58^ (*n* = 71,768; gray). For **a**–**d**, calculations assume no net air–sea CO_2_ change. Best fits of data are shown by red curves. See Methods for calculation details

**Fig. 5:**
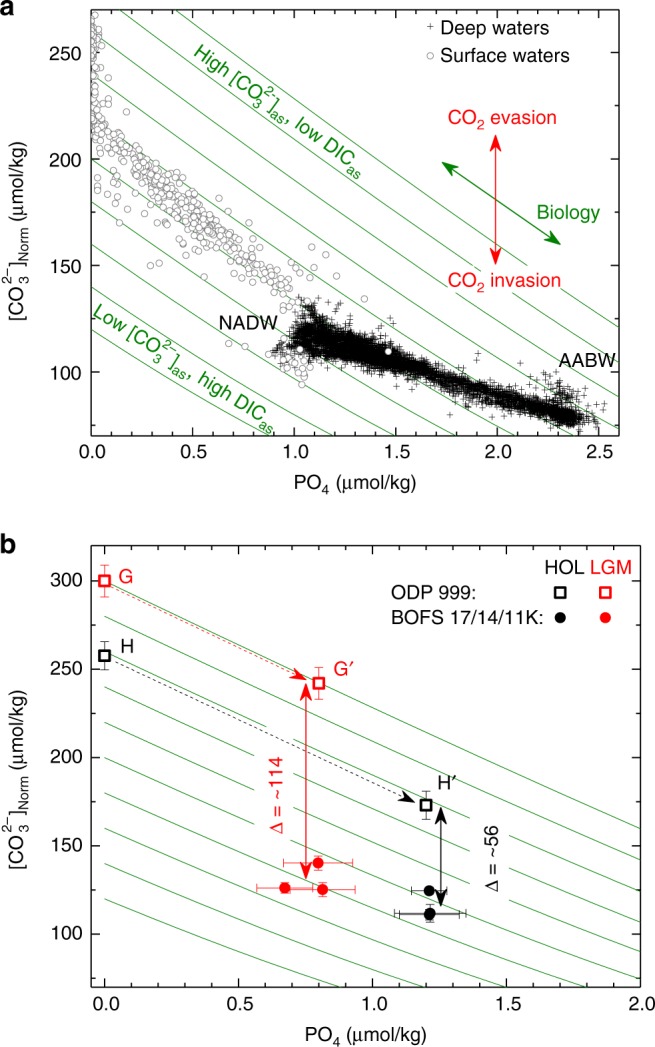
[CO_3_^2−^]_Norm_ vs. PO_4_. **a** Preindustrial Atlantic surface (<100 m, north of 10°N) and deep (>1000 m, 65°N–65°S) water data^2^. **b** Holocene and LGM data. Error bars, 2*σ*

Surface-water [CO_3_^2−^] at ODP 999 is ~150 μmol/kg higher than deep-water values at BOFS cores (Fig. 3), but this [CO_3_^2−^] contrast includes influences from physical (via dissociation constants) and biological (via within-ocean DIC and ALK redistributions) changes in addition to any air–sea CO_2_ changes between surface and deep waters. Below, we present a pragmatic recipe to estimate [CO_3_^2^^−^]_as_ gradients between water masses. We take advantage of well-defined sensitivities of [CO_3_^2−^] to T–S–P (Fig. 4) to calculate normalized seawater [CO_3_^2−^] ([CO_3_^2−^]_Norm_) at conditions of *T* = 3 °C, *S* = 35‰, and *P* = 2500 dbar (Methods). Any variation in T–S–P would affect seawater [CO_3_^2−^] via (i) changing CO_2_ system dissociation constants, and (ii) altering the solubility pump and thereby air–sea exchange component CO_2_ concentrations in seawater. Calculation of [CO_3_^2^^−^]_Norm_ only corrects for influences from (i), without affecting any air–sea CO_2_ signal. After normalization to constant T–S–P conditions and assuming no net air–sea exchange, biological activity drives changes in both [CO_3_^2−^]_Norm_ and PO_4_ along the biological trend (green curves in Fig. 5; Methods). Note that along a certain biological trend, seawater [CO_3_^2^^−^]_Norm_ and PO_4_ are only affected by within-ocean DIC and ALK redistributions (Fig. 1b). A net air–sea CO_2_ change would cause changes in [CO_3_^2−^]_Norm_ and PO_4_ across biological curves. At the same PO_4_, [CO_3_^2−^]_Norm_ contrasts reflect [CO_3_^2−^]_as_ gradients due to air–sea CO_2_ exchange between water masses.

A plot of [CO_3_^2−^]_Norm_ vs. PO_4_ greatly facilitates investigation of air–sea CO_2_ exchange from combined [CO_3_^2^^−^] and PO_4_ measurements/reconstructions. Compared to the biological trend, preindustrial North Atlantic surface waters have a steeper trend (Fig. 5a), which reflects CO_2_ absorption during northward transport. Deep-water data lie on a shallower trend, consistent with mixing between low-[CO_3_^2−^]_as_ (high DIC_as_) NADW and high-[CO_3_^2^^−^]_as_ (low DIC_as_) AABW in the deep Atlantic (Fig. 2).

For our downcore reconstructions, benthic Cd/Ca suggests that deep-waters at the BOFS sites had PO_4_ values of ~1.2 and ~0.8 μmol/kg during the Holocene and LGM, respectively (Fig. 5b; Supplementary Fig. 8). Assuming no air–sea CO_2_ exchange, [CO_3_^2−^]_Norm_ of ODP 999 surface waters at elevated PO_4_ due to biological processes can be estimated straightforwardly using the H→H′ and G→G′ trajectories in Fig. 5b for the Holocene and LGM, respectively. For the Holocene, ODP 999 [CO_3_^2−^]_Norm_ is ~56 ± 8 μmol/kg higher than [CO_3_^2−^]_Norm_ of BOFS cores at PO_4_ = 1.2 μmol/kg. For the LGM, ODP 999 [CO_3_^2−^]_Norm_ is ~114 ± 9 μmol/kg higher than [CO_3_^2−^]_Norm_ of BOFS cores at PO_4_ = 0.8 μmol/kg. This suggests a Holocene-to-LGM increase of ~58 ± 12 μmol/kg in the ODP 999−BOFS [CO_3_^2−^]_as_ gradient.

We also present a second approach to calculate [CO_3_^2−^]_as_ gradients, which involves frequent use of the CO_2_sys program^28^ and intermediate-step ALK and DIC parameters (Supplementary Note 1; Supplementary Figs. 9 and 10). The approach gives similar results as the above pragmatic recipe, because both methods are essentially based on the same principle, which is to compare [CO_3_^2−^] of water masses at the same physical and biological conditions.

### Enhanced CO_2_ uptake in the glacial North Atlantic

What caused the greater ODP 999−BOFS [CO_3_^2−^]_as_ gradient during the LGM? We consider influences from biogenic matter composition variations, surface-water ALK and PO_4_ changes, ocean circulation changes, and North Atlantic air–sea exchange. In Fig. 5, we have used a soft-tissue Redfield C/PO_4_ of 127 and a rain ratio (*R*, = C_organic_:${\mathrm{C}}_{{{\rm{CaCO}}_3}}$) of 4 (refs. ^3,10,29,30^) to predict the biological trend. Raising LGM C/PO_4_ to 140 (the high end value in today’s North Atlantic^30^) and *R* to 8 (doubling of the modern value) could lower the LGM [CO_3_^2−^]_as_ gradient by ~16 μmol/kg (Supplementary Fig. 15), still leaving ~42 μmol/kg [CO_3_^2−^]_as_ gradient increase to be explained by other processes. Evidence for such large biological changes is lacking. Importantly, any increase in C/PO_4_ and *R* would implicitly sequester more atmospheric CO_2_ via an enhanced soft-tissue pump and weakened carbonate pump^15^. Inclusion of a whole ocean ALK inventory change^6^ or any increased glacial surface-water PO_4_ at ODP 999 would raise the LGM [CO_3_^2−^]_as_ gradient (Supplementary Figs. 16 and 17).

Regarding ocean circulation changes, most AAIW upwells in the tropics and less than ~25% of today’s NADW is fed directly by AAIW without surfacing at low latitudes^17^. Northward AAIW transport is thought to have been reduced substantially in the glacial Atlantic^23,31–33^ in the face of vigorous GNAIW production^34^. Assuming a constant total carbon uptake by the North Atlantic, a complete shutdown of AAIW contribution would only raise the ODP 999−BOFS [CO_3_^2−^]_as_ gradient by ~30%, which is much smaller than the ~100% increase from the Holocene (~56 μmol/kg) to LGM (~114 μmol/kg) (Fig. 5b). Any increased mixing of glacial AABW at BOFS sites would reduce the ODP 999−BOFS [CO_3_^2−^]_as_ gradient during the LGM. Given the proximity of our deep-water sites to the core of GNAIW and Nordic Sea overflow waters^23,24,31,35,36^, the larger LGM [CO_3_^2−^]_as_ gradient between ODP 999 and BOFS cores likely reflects a greater DIC_as_ increase from Gulf Stream to GNAIW. North Atlantic CO_2_ invasion was responsible for the preindustrial Gulf Stream-NADW [CO_3_^2^^−^]_as_ gradient (Fig. 2). Therefore, we ascribe the increased ODP 999−BOFS [CO_3_^2−^]_as_ gradient during the LGM to more efficient atmospheric CO_2_ uptake via air–sea exchange and subsequent transport to at least ~2 km depth (BOFS 11 K core depth) in the glacial North Atlantic.

### Quantification of North Atlantic CO_2_ uptake

With reconstructed ODP 999−BOFS [CO_3_^2−^]_as_ gradients, we further quantify North Atlantic air–sea CO_2_ absorption changes between the Holocene and LGM. [CO_3_^2−^]_as_/DIC_as_ sensitivities can be precisely estimated (Fig. 4e), making [CO_3_^2−^]_as_ gradients a useful proxy to calculate DIC_as_ changes. The 58 ± 12 μmol/kg Holocene-to-LGM [CO_3_^2−^]_as_ increase (Fig. 5b) indicates a DIC_as_ increase of 91 ± 20 μmol/kg due to enhanced North Atlantic air–sea CO_2_ absorption (Methods). Compared to the preindustrial Gulf Stream-NADW DIC_as_ gradient of ~90 μmol/kg (Fig. 2, Supplementary Fig. 3), this suggests a doubling of CO_2_ uptake efficiency in the LGM North Atlantic.

**Fig. 6:**
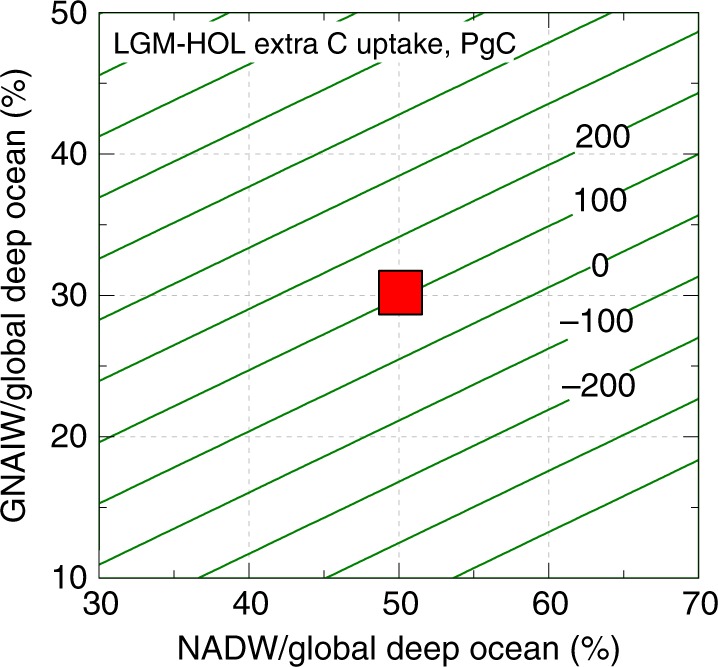
North Atlantic CO_2_ budget. The LGM–Holocene extra carbon uptake is based on Holocene-to-LGM DIC_as_ increase of 91 μmol/kg. The large red square represents our best estimate of ~100 PgC, assuming that NADW and GNAIW occupied ~50% and ~30% of the global deep ocean (>1 km), respectively^35,36,38,39^. See Methods for calculation details

Beside DIC_as_ gradient changes, which indicate air–sea CO_2_ uptake efficiency, knowledge of northern-sourced-water volumes in the global deep ocean is required to determine total North Atlantic carbon sequestration. Figure 6 shows the total extra carbon absorbed by the LGM North Atlantic for a range of northern-sourced-water volumes (Methods). Sedimentary Pa/Th, radiocarbon, neodymium isotopes, and paired benthic Cd/Ca–δ^13^C suggest^32,34,35,37^ vigorous glacial northern-sourced intermediate water production and subsequent transport to the remaining world ocean. Based on previous estimates^35,36,38,39^, we tentatively assume that NADW- and GNAIW-derived waters occupy ~50% and ~30%, respectively, of the global deep ocean volume (1 × 10^18^ m^3^ for >1 km). In this case, our ~91 μmol/kg Holocene-to-LGM DIC_as_ increase yields ~100 Petagrams of carbon (PgC; 1 Pg = 1 × 10^15^ g) greater CO_2_ sequestration by the LGM North Atlantic (Fig. 6; Methods). To maintain similar total carbon uptake between the Holocene and LGM, GNAIW would need to be less than ~50% of NADW in volume, which we consider unlikely given evidence for intensive GNAIW export to the global ocean^23,32,34,35,37^. We acknowledge uncertainties associated with our calculations, and encourage future work to better constrain volumes and carbonate chemistry changes of various water masses in the past.

## Discussion

Previous work^40–42^ has tried to constrain air–sea CO_2_ exchange by reconstructing surface conditions. This requires reconstructions of the air–sea *p*CO_2_ difference (influenced by T, S, and nutrient utilization), the gas transfer velocity (a power function of wind speed), solubility of CO_2_ in seawater (mainly affected by T), and the area and contact time of surface waters available for air–sea exchange^1^. Sea ice cover^43^ possibly expanded, reducing glacial North Atlantic CO_2_ absorption. A larger LGM meridional surface temperature gradient^43,44^ would enhance the North Atlantic solubility pump^13^. Existing planktonic δ^15^N and Cd/Ca data^40,45^ show conflicting results regarding the glacial North Atlantic nutrient conditions, perhaps due to complications associated with surface-water proxies and spatial/seasonal nutrient variations in the North Atlantic. A decreased preformed nutrient in the glacial North Atlantic might be inferred from a lower GNAIW PO_4_ (Fig. 3), but faster ventilation and/or reduced glacial AAIW could also cause a nutrient decline in GNAIW^23,34,46^. Little is known about past wind intensity and air–sea contact time changes. Consequently, potential North Atlantic glacial CO_2_ invasion remains poorly understood. Bypassing the necessity to reconstruct surface-water conditions for which some proxies are still lacking (e.g., wind), our new approach, to our knowledge, offers the first proxy-based quantitative estimate of air–sea CO_2_ uptake efficiency in the glacial North Atlantic.

In contrast to previous calculations^47–49^ which concern combined biological (i.e., within-ocean DIC redistribution) and air–sea exchange carbon changes (Fig. 1c), our total North Atlantic carbon uptake estimate only represents the net air–sea CO_2_ change that is more directly relevant to atmospheric and terrestrial carbon inventory variations. Our estimated ~100 PgC sequestration constitutes ~15% of the Holocene-LGM ~600 PgC change associated with the atmosphere (~200 PgC) and terrestrial biosphere (~400 PgC)^5,6^. Given this global carbon budget context, our work reinforces the role of other polar regions (e.g., Southern Ocean) in controlling the glacial–interglacial carbon cycle. However, if there were no efficiency enhancement for the LGM North Atlantic, a 40% shrinkage of NADW volume would decrease air–sea component CO_2_ sequestration by ~240 PgC in the deep ocean (Methods). Therefore, by overcoming this opposing “volume effect”, the improved glacial North Atlantic efficiency increased DIC_as_ values of northern-sourced deep waters (termed the “endmember effect”) and thereby contributed substantially to air–sea CO_2_ sequestration in the LGM deep ocean.

Atmospheric *p*CO_2_ is controlled by both CO_2_ gains (e.g., via Southern Ocean outgassing) and losses (e.g., via North Atlantic absorption)^2,3,11^. Growing evidence indicates that processes outside the Southern Ocean may have affected past atmospheric CO_2_ variations^50–52^. Our proxy-based results indicate that the North Atlantic CO_2_ pump efficiency during the LGM was almost doubled relative to the Holocene. This increased efficiency and associated “endmember effect” effectively outcompeted the opposing “volume effect” due to any shrinkage of northern-sourced deep waters in the world ocean. In addition to the well-recognized role of reduced outgassing in the Southern Ocean^6,8,9,47,53,54^, we therefore suggest that variations in the uptake and sequestration of atmospheric CO_2_ via the North Atlantic Ocean were important contributors to glacial/interglacial carbon cycling.

## Methods

### CO_2_ system calculations

For both the preindustrial ocean and down-core CO_2_ system calculations, seawater carbonate system variables were calculated using the CO_2_sys.xls program^28^ with dissociation constants *K*_1_ and *K*_2_ according to Mehrbach et al.^55^ and ${K}_{{{\mathrm{SO}}_4}}$ according to Dickson^56^. Seawater total boron concentration was calculated from the boron–salinity relationship of Lee et al.^57^. For the GLODAP dataset, the anthropogenic CO_2_ contribution was subtracted from the measured DIC to obtain preindustrial DIC values^2^.

### Preindustrial Atlantic DIC_as_ and [CO_3_^2−^]_as_

The GLODAP dataset^2^ is used to calculate preindustrial ocean CO_2_ system variables. Following the established method of Broecker and Peng^3^, we account for DIC anomalies created by (1) freshwater addition or removal based on S, (2) soft-tissue carbon creation and respiration based on PO_4_, and (3) CaCO_3_ formation and dissolution based on ALK and nitrate (NO_3_). See Fig. 1 for the simplified concept. We adopt the term DIC_as_ to represent net air–sea exchange component DIC signatures from:

1 $$ {\hskip-10pt}{{\mathrm{DIC}}_{\rm{as}}} = {{\mathrm{DIC}}_{\rm{s}}} - ({{\mathrm{PO}}_{\rm{4s}}} 	- {{{\mathrm{{PO}}}_4}^{\rm{mo}}}) \times {{\mathrm{C/PO}}_4}\\ 	- {\ }^{1}{\hskip-2.5pt} {\hbox{/}}{\ }_{{\hskip-5pt} 2}\times \left( {{\mathrm{ALK}}_{\mathrm{s}}-{\mathrm{ALK}}^{{\mathrm{mo}}} + {\mathrm{NO}}_{{\mathrm{3s}}}-{{\mathrm{{NO}}}_3}^{\rm{mo}}} \right)-{\mathrm{DIC}}_{{\mathrm{constant}}}$$

where the subscript “s” represents values normalized to *S* of 35 (e.g., DIC_s_ = DIC × 35/*S*); the superscript “mo” denotes mean ocean values at *S* = 35 (PO_4_^mo^ = 2.2 μmol/kg, ALK^mo^ = 2383 μmol/kg, DIC^mo^ = 2267 μmol/kg, and NO_3_^mo^ = 31 μmol/kg)^29^; C/PO_4_ represents the soft-tissue stoichiometric Redfield ratio; and the arbitrary DIC_constant_ ( = 2285 μmol/kg) is designed to bring zero DIC_as_ close to the NADW–AABW boundary (Fig. 2). The term (PO_4s_ − PO_4_^mo^) × C/PO_4_ corrects for DIC changes due to photosynthesis and soft-tissue degradation, and the term ½ × (ALK_s_ − ALK^mo^ + NO_3s_ − NO_3_^mo^) accounts for DIC changes caused by CaCO_3_ formation and dissolution. To be consistent with previous work^3,30^, we used C/PO_4_ = 127 to calculate DIC_as_ and [CO_3_^2−^]_as_ in Fig. 2. Using other C/PO_4_ values^10^ does not significantly affect spatial DIC_as_ and [CO_3_^2^^−^]_as_ patterns (Supplementary Figs. 11 and 12). Neither are their patterns affected by using other PO_4_–ALK–NO_3_ values to replace global mean values in Eq. (1) (Supplementary Figs. 13 and 14). Ideally, DIC_constant_ would be the mean DIC value of an abiotic ocean (Fig. 1), but this value cannot be simply determined from modern observations. Because our interest lies in spatial DIC_as_ contrasts instead of absolute values, the choice of DIC_constant_ has no effect on our interpretation.

To obtain [CO_3_^2−^]_as_, we first calculate [CO_3_^2^^−^]_PO4–T–S–P_ using (DIC_as_ + DIC_constant_), ALK^mo^, and PO_4_^mo^ at *T* = 3 °C, *S* = 35, and *P* = 2500 dbar. [CO_3_^2−^]_as_ is then calculated by [CO_3_^2^^−^]_as_ = [CO_3_^2^^−^]_PO4–T–S–P_ − [CO_3_^2−^]_constant_, where [CO_3_^2^^−^]_constant_ ( = 78 μmol/kg, calculated using DIC_constant_ and ALK^mo^) is designed to bring zero [CO_3_^2−^]_as_ close to the NADW–AABW boundary. In essence, the [CO_3_^2−^]_as_ distribution reflects the variation of [CO_3_^2−^] when normalized to the same PO_4_−T−S−P conditions.

### CO_2_ system sensitivities and calculation of [CO_3_^2^^−^]_Norm_

Because the seawater CO_2_ system is nonlinear, there is currently no simple way to derive these sensitivities based on CO_2_ system equations^16^. We use GLODAP preindustrial data^2^ to calculate numerically [CO_3_^2−^] sensitivities to various physiochemical parameters. Use of LGM outputs from the LOVECLIM model^58^ yields comparable sensitivities. We first use hydrographic data, including T, S, P, DIC, ALK, PO_4_, and SiO_3_ to calculate [CO_3_^2−^]. We then change *S* to 35‰ and other chemical concentrations proportionally. For example, ALK and DIC will change as follows:

2 $${\mathrm{ALK}}_{{\mathrm{s = 35}}}={\mathrm{ALK} \,\times 35/S,}\,{\mathrm{and}}$$

3 $${\mathrm{DIC}}_{{\mathrm{s = 35}}} = {\mathrm{DIC}} \; \times {{35/S}}{.}$$

We use *S* = 35‰, ALK_S=35_, DIC_S=35_, [PO_4_]_S=35_, and [SiO_3_]_S=35_ along with hydrographic T and P to calculate [CO_3_^2−^]_S=35_. The [CO_3_^2^^−^] to S sensitivity (Sen__S_) is calculated by:

4 $${\mathrm{Sen}}_{\_{\mathrm{S}}} = \left( {\left[ {{{\mathrm{CO}}_{\mathrm{3}}}^{{\mathrm{2 - }}}} \right]-\left[ {{{\mathrm{CO}}_{\mathrm{3}}}^{{\mathrm{2 - }}}} \right]_{{\mathrm{S = 35}}}} \right)/\left( {{S}-{\mathrm{35}}} \right){.}$$

To estimate temperature effects, we calculate [CO_3_^2−^]_S=35, T=3 °C_ using *S* = 35‰, ALK_S=35_, DIC_S=35_, [PO_4_]_S=35_, [SiO_3_]_S=35_, *T* = 3 °C, and hydrographic P. The sensitivity of [CO_3_^2−^]_S=35_ to temperature (Sen__T_) is defined by:

5 $${\mathrm{Sen}}_{\_{\mathrm{T}}}\,{\mathrm{ = }}\,\left( {\left[ {{{\mathrm{CO}}_{\mathrm{3}}}^{{\mathrm{2 - }}}} \right]_{{\mathrm{S = 35,}}\,{\mathrm{T}} = {\mathrm{3}}^ {\circ} {\mathrm{C}}}-\left[ {{{\mathrm{CO}}_{\mathrm{3}}}^{{\mathrm{2 - }}}} \right]_{{\mathrm{S}} = {\mathrm{35}}}} \right)/\left( {{\mathrm{3}}-{T}} \right){.}$$

Regarding pressure effects, we calculate [CO_3_^2−^]_S=35, T=3 °C, P=2500 dbar_ using *S* = 35‰, ALK_S=35_, DIC_S=35_, [PO_4_]_S=35_, [SiO_3_]_S=35_, *T* = 3°C, and *P* = 2500 dbar. The sensitivity of [CO_3_^2−^]_S=35, T=3°C_ to P (Sen__P_) is defined by:

6 $${\mathrm{Sen}}_{\_{\mathrm{P}}} =	 \,\left( {\left[ {{{\mathrm{CO}}_{\mathrm{3}}}^{{\mathrm{2 - }}}} \right]_{{\mathrm{S= 35, }}\,{\mathrm{T}} = {\mathrm{3}}^ {\circ} {\mathrm{C,}}\,{\mathrm{P}} = {\mathrm{2500}}\,{\mathrm{dbar}}}} \right. \\ 	\left. {-\left[ {{{\mathrm{CO}}_{\mathrm{3}}}^{{\mathrm{2 - }}}} \right]_{{\mathrm{S}} = {\mathrm{35,}}\,{\mathrm{T}} = {\mathrm{3}}^ {\circ} {\mathrm{C}}}} \right)/\left( {{\mathrm{2500}}-{P}} \right){\mathrm{ \times 100}}.$$

To estimate the influence on [CO_3_^2−^] from within-ocean ALK–DIC redistributions by biological processes, we assume a 0.1 μmol/kg increase in PO_4_ (i.e., ΔPO_4_ = 0.1 μmol/kg) due to biological respiration (photosynthesis has an opposite effect). The resultant ALK (ALK_S=35+respiration_) and DIC (DIC_S=35+respiration_) can then be calculated from:

7 $${\mathrm{ALK}}_{{\mathrm{S}} = {\mathrm{35}} + {\mathrm{respiration}}} =	 {\,} {\mathrm{ALK}}_{{\mathrm{s = 35}}} + {\mathrm{\Delta PO}}_{\mathrm{4}} \times {\mathrm{C/PO}}_{\mathrm{4}}\\ 	\div \, {R} \times {\mathrm{2}}-{\mathrm{\Delta PO}}_{\mathrm{4}} \times {\mathrm{N/PO}}_{\mathrm{4}}.$$

8 $${\mathrm{DIC}}_{{\mathrm{S}} = {\mathrm{35}} + {\mathrm{respiration}}} = {\mathrm{DIC}}_{{\mathrm{s}} = {\mathrm{35}}} + {\mathrm{\Delta PO}}_{{\mathrm{4}}} \times {\mathrm{C/PO}}_{\mathrm{4}} + {\mathrm{\Delta PO}}_{{\mathrm{4}}} \times {\mathrm{C/PO}}_{\mathrm{4}} \div {R}{.}$$

Resultant [CO_3_^2−^] ([CO_3_^2^^−^]_Norm+respiration_) values are calculated using DIC_S=35+respiration_, ALK_S=35+respiration_, and ([PO_4_]_S=35_ + ΔPO_4_) at constant physical conditions of *T* = 3 °C, *S* = 35, and *P* = 2500 dbar. The sensitivity of [CO_3_^2^^−^]_Norm_ to PO_4_ is defined by:

9 $$	\left[ {{{\mathrm{CO}}_{\mathrm{3}}}^{{\mathrm{2 - }}}} \right]_{{\mathrm{Norm}}}{\mathrm{/PO}}_{\mathrm{4}}\,{\mathrm{sensitivity}} = \\ 	\left( {\left[ {{{\mathrm{CO}}_{\mathrm{3}}}^{{\mathrm{2 - }}}} \right]_{{\mathrm{Norm}} + {\mathrm{respiration}}}{\mathrm{- }}\left[ {{{\mathrm{CO}}_{\mathrm{3}}}^{{\mathrm{2 - }}}} \right]_{{\mathrm{Norm}}}} \right)/{\mathrm{\Delta PO}}_{\mathrm{4}}.$$

We consider four Redfield stoichiometric scenarios: C/PO_4_ = 127, *R* = 4 (the reference composition; Fig. 4d); C/PO_4_ = 140, *R* = 4; C/PO_4_ = 127, *R* = 8; and C/PO_4_ = 140, *R* = 8 (Supplementary Fig. 15). In all cases, strong exponential correlations exist between [CO_3_^2−^]_Norm_/PO_4_ sensitivities and [CO_3_^2−^]_Norm_. The correlations may reflect the buffering effect of the seawater CO_2_ system: for seawater with high DIC (low [CO_3_^2^^−^] and high buffering capability), [CO_3_^2−^] would be relatively less sensitive to biological DIC and ALK disturbances. All of the above sensitivity calculations assume no net air–sea CO_2_ change.

To calculate air–sea exchange sensitivities, we assume a 10 μmol/kg increase in DIC_S=35_ due to atmospheric CO_2_ invasion (i.e., ΔDIC_as_ = 10 μmol/kg). We calculate [CO_3_^2−^]_Norm+as_ using *S* = 35‰, ALK_S=35_, DIC_S=35+as_ ( = DIC_S=35_ + ΔDIC_as_), [PO_4_]_S=35_, [SiO_3_]_S=35_, *T* = 3 °C, and *P* = 2500 dbar. The sensitivity of [CO_3_^2−^]_as_ to DIC_as_ is defined by:

10 $$	\left[ {{{\mathrm{CO}}_{\mathrm{3}}}^{{\mathrm{2 - }}}} \right]_{{\mathrm{as}}}{\mathrm{/DIC}}_{{\mathrm{as}}}\,{\mathrm{sensitivity}} = \\ 	\left( {\left[ {{{\mathrm{CO}}_{\mathrm{3}}}^{{\mathrm{2 - }}}} \right]_{{\mathrm{Norm}} + {\mathrm{as}}}-\left[ {{{\mathrm{CO}}_{\mathrm{3}}}^{{\mathrm{2 - }}}} \right]_{{\mathrm{Norm}}}} \right)/\Delta {\mathrm{DIC}}_{{\mathrm{as}}}.$$

Using sensitivities shown in Fig. 4, [CO_3_^2^^−^]_Norm_ can be calculated by:

11 $$\left[ {{{\mathrm{CO}}_{\mathrm{3}}}^{{\mathrm{2 - }}}} \right]_{{\mathrm{Norm}}} \,=	 \,\left[ {{{\mathrm{CO}}_{\mathrm{3}}}^{{\mathrm{2 - }}}} \right] + \left( {{\mathrm{35}}-{S}} \right) \times {{\mathrm{Sen}}_{\_{\mathrm{S}}}} + \left( {{\mathrm{3}}-{T}} \right)\\ 	\times \, {{\mathrm{Sen}}_{\_{\mathrm{T}}}} + \left( {{\mathrm{2500}}-{P}} \right){\mathrm{/100}} {\hskip1pt}\times {{\mathrm{Sen}}_{\_{\mathrm{P}}}}.$$

Excel spreadsheets are provided in Supplementary Data 7–8 to calculate [CO_3_^2−^]_Norm_ and the biological curves shown in Fig. 5.

### LGM–Holocene North Atlantic carbon budget

The total extra carbon increase (Δ∑C_LGM−Holocene_) in Fig. 6 is calculated by Δ∑C_LGM−Holocene_ = *V *× density × %GNAIW × ([CO_3_^2−^]_as_ODP999-BOFS_^LGM^/0.61) × 12 − *V *× density × %NADW × ([CO_3_^2^^−^]_as_ODP999-BOFS_^Holocene^/0.59) × 12, where *V* is the global deep ocean volume (>1 km water depth) at 100.8 × 10^16^ m^3^, density = 1027.8 kg/m^3^ (ref. ^29^), %GNAIW and %NADW, respectively, represent their volume fractions in the deep ocean, [CO_3_^2^^−^]_as_ODP999-BOFS_^Holocene^ = 56 μmol/kg, [CO_3_^2^^−^]_as_ODP999-BOFS_^LGM^ = 114 μmol/kg (Fig. 5), terms 0.61 and 0.58, respectively, represent the absolute LGM and Holocene [CO_3_^2−^]_as_/DIC_as_ sensitivities (Fig. 4e) used to transfer [CO_3_^2−^]_as_ODP999-BOFS_ into ODP999–BOFS DIC_as_ contrasts (LGM: 186 μmol/kg; Holocene: 95 μmol/kg), and the number 12 converts C from moles into weight. Based on previous estimates, %NADW is thought to be ~50% (refs. ^38,39^), while %GNAIW remained roughly similar to %NADW or shrank (refs. ^35,36^). These estimates are debated and have large uncertainties, and we thus calculate Δ∑C_LGM−Holocene_ for a range of %NADW and %GNAIW values (Fig. 6). Any influence from AAIW is ignored because of its similar [CO_3_^2−^]_as_ signals to Gulf Stream during the Holocene (Supplementary Fig. 3) and much reduced northward advection during the LGM^23,31–33^. We tentatively treat Δ∑C_LGM-Holocene_ of ~100 PgC using %NADW = 50% and %GNAIW = 30% as our best estimate. Assuming no Holocene–LGM DIC_as_ gradient change (i.e., the same CO_2_ uptake efficiency) and everything else being equal, Δ∑C_LGM–Holocene_ would be −240 PgC at %NADW = 50% and %GNAIW = 30%.

### Cores, age models, samples, and analytical methods

We used ODP Site 999 for Gulf Stream surface-water reconstructions (Fig. 2). The age model is from Schmidt et al.^59^. Planktonic foraminiferal *Globigerinoides ruber* (*sensu stricto*, white variety) δ^18^O, Mg/Ca, and δ^11^B data are from refs. ^21,22,59^. Briefly, about 25 and 55 shells from the 250–350 μm size fraction were used for δ^18^O and Mg/Ca analyses, respectively. Samples for δ^18^O analyses were sonicated in methanol for 5–10 s, roasted under vacuum at 375 ^o^C for 30 min, and analyzed on a Fisons Optima IRMS with a precision of <0.06‰. Shells for Mg/Ca were cleaned following the reductive cleaning procedure^60^ and measured on an inductively-coupled plasma mass spectrometer (ICP–MS) with a precision of ~1.7%. For δ^11^B analyses, about 100–120 *G. ruber* (w) shells from the 300–355 μm size fraction were cleaned following the “Mg-cleaning” procedure^61^, to minimize material loss during cleaning^62^. *G. ruber* (w) δ^11^B was measured on a Neptune multicollector (MC)–ICP–MS with an analytical error in δ^11^B of about ±0.25‰ (ref. ^21^).

Three cores (BOFS 17, BOFS 11, and BOFS 14 K) from the polar North Atlantic Ocean are used for deep-water reconstructions (Fig. 3). Their age models are based on published chronologies^24,63–65^. For each sample (~2 cm thickness), ~10–20 cm^3^ of sediment was disaggregated in de-ionized water and was wet sieved through 63 μm sieves. To facilitate analyses, we picked the most abundant species for measurements. For each B/Ca analysis, ~10–20 monospecific shells of the benthic foraminifera *C. mundulus* (BOFS 17 K) and *C. wuellerstorfi* (BOFS 14, 11 K) were obtained from 250 to 500 μm size fraction. The shells were double checked under a microscope before crushing to ensure that consistent morphologies were used throughout the core. On average, following this careful screening the starting material for each sample was ~8–12 shells, which is equivalent to ~300–600 μg of carbonate. For benthic B/Ca analyses, foraminiferal shells were cleaned with either the “Mg-cleaning” method^61^ or the “Cd-cleaning” protocol^61^, to investigate cleaning effects on trace element/Ca in foraminiferal shells^62,66^. No discernable B/Ca difference is observed between the two cleaning methods^25,62^. Benthic B/Ca ratios were measured on an ICP–MS using procedures outlined in ref. ^67^, with an analytical error better than ~5%.

For each benthic Cd/Ca analysis, ~10–20 shells of the benthic foraminiferal taxa *C. mundulus* (BOFS 17 K), *C. wuellerstorfi* (BOFS 14 K, 11 K), and *Uvigerina* spp. (BOFS 17 K) were picked from the 250–500 μm size fraction. Previous studies^26,27,68^ showed similar Cd/Ca ratios between infaunal *Uvigerina* spp. and epifaunal *Cibicidoides*, and we thus combined Cd/Ca data from these taxa to obtain continuous downcore PO_4_ records. We used the “Cd-cleaning” method^60,69^ to clean benthic shells for Cd/Ca measurements. Cd/Ca ratios were measured on an ICP–MS with an analytical error better than ~5% (ref. ^67^)

For δ^11^B measurements, about 20 benthic shells from the 250–500 μm size fraction were picked for each sample. Shells used for δ^11^B analyses were cleaned using the “Mg-cleaning” method, to minimize loss of shell material^61^. After cleaning, shells were dissolved and pure boron was extracted using column chemistry as described by Foster^21^. Benthic δ^11^B was measured on a Neptune multi-collector (MC)–ICP–MS following ref. ^21^. The analytical error in δ^11^B is about ± 0.25‰. Due to the relatively large sample size requirement, shell availability, and lengthy chemical treatments for δ^11^B, we present low-resolution δ^11^B for *C. mundulus* from BOFS 17 K and for *C. wuellerstorfi* from BOFS 11 K. Note that consistent [CO_3_^2−^] results from B/Ca and δ^11^B strengthen the reliability of our reconstructions (Fig. 3).

Published benthic Cd/Ca and B/Ca results are included in Fig. 3. Altogether, we generated 180 new measurements of benthic δ^11^B, B/Ca, and Cd/Ca. All data are listed in Supplementary Data 1–9.

### ODP 999 reconstructions

ODP Site 999 was used to constrain past physical conditions and carbonate chemistry of the Gulf Stream (Supplementary Fig. 4). Following previous approaches^21,22^, surface water temperature (T_surface_) and salinity (S_surface_) were estimated based on *G. ruber* Mg/Ca (ref. ^59^) and sea level changes^21,22,59^, respectively. We first convert *G. ruber* δ^11^B to borate δ^11^B (δ^11^B_borate_), following the conversion method of ref. ^22^. Surface water pH (pH_surface_) was calculated from seawater δ^11^B_borate_ along with T_surface_ and S_surface_. To constrain the CO_2_ system, two CO_2_ system variables are necessary^16^. In addition to δ^11^B-derived pH, literature studies^21,22,41^ generally estimate past surface-water ALK (ALK_surface_) changes. Following refs. ^21,22^, we estimate ALK_surface_ from S_surface_ using the modern S_surface_–ALK_surface_ relationship (ALK_surface_ = 59.19 × *S*_surface_ + 229.08, *R*^2^ = 0.99)^21^. Together with T_surface_ and S_surface_, pH_surface_, and ALK_surface_ were used to calculate other CO_2_ system variables including surface-water [CO_3_^2^^−^] ([CO_3_^2−^]_surface_) and DIC (DIC_surface_) using the CO_2_sys program^28^. Surface-water PO_4_ concentration at ODP 999 is assumed to be zero over the last 27 ka.

Following refs. ^21,22,59^, errors are estimated to be 1 °C, 1‰, 100 μmol/kg, and ~0.43‰ for T_surface_, S_surface_, ALK_surface_, and δ^11^B_borate_, respectively. Integrated average uncertainties in [CO_3_^2−^]_surface_ and DIC_surface_ for a single reconstruction are, respectively, ~20 (Holocene: ~18, LGM: ~24) and ~90 μmol/kg, based on quadratic addition of all individual errors sourced from T_surface_ ([CO_3_^2−^]_surface_: 2 μmol/kg, DIC_surface_: 3 μmol/kg), S_surface_ ([CO_3_^2−^]_surface_: 2 μmol/kg, DIC_surface_: 5 μmol/kg), ALK_surface_ ([CO_3_^2−^]_surface_: 14 μmol/kg, DIC_surface_: 86 μmol/kg), and δ^11^B_borate_ ([CO_3_^2^^−^]_surface_: 16 μmol/kg, DIC_surface_: 24 μmol/kg; note that δ^11^B_borate_ leads to an error in [CO_3_^2−^] via pH). Uncertainties for calculated CO_2_ system variables at ODP 999 are tabulated in Supplementary Data 1. Use of other methods to estimate ALK would have little impact on our conclusions (Supplementary Figs. 16 and 17).

### From pH to [CO_3_^2−^]

For palaeo-studies, surface-water pH is generally obtained from planktonic foraminiferal δ^11^B. To calculate [CO_3_^2−^], a second CO_2_ system variable is needed^16^. Following the previous approach^21,22^, past ALK_surface_ at ODP 999 have been estimated from S using the S_surface_–ALK_surface_ relationship. Due to limited knowledge about the past S_surface_–ALK_surface_ relationship, a generous uncertainty has been assigned to ALK_surface_ at ±100 μmol/kg (ref. ^21,22^), which is about half of the entire ALK range in the present global ocean^2^. Using ALK_surface_ and pH_surface_ along with T_surface_ and S_surface_, [CO_3_^2−^]_surface_ and DIC_surface_ can be calculated using the CO_2_sys program^28^. Because of the large uncertainty in ALK_surface_, large errors in DIC_surface_ might be expected (Supplementary Fig. 4). However, given the constraint from pH_surface_, seawater ALK_surface_ and DIC_surface_ variations are not random but must vary systematically within ALK–DIC space (Supplementary Fig. 5). Because of the close relationship between pH and [CO_3_^2−^] (i.e., roughly parallel patterns of pH and [CO_3_^2−^] within ALK−DIC space; Supplementary Fig. 5), this systematic ALK−DIC variation allows us to confine [CO_3_^2^^−^] with acceptable uncertainty. For a given pH at ODP 999, an error of 100 μmol/kg in ALK only leads to an error of about ±14 μmol/kg in [CO_3_^2^^−^] (Supplementary Fig. 5).

For clarity, Supplementary Fig. 5a, b only consider the effect of ALK errors on [CO_3_^2^^−^] estimates assuming constant pH and T–S–P conditions. To fully propagate errors from various sources including T_surface_, S_surface_, ALK_surface_, and pH_surface_, we use a Monte Carlo approach (*n* = 10,000) to calculate the integrated error in [CO_3_^2−^] (ref. ^70^). As can be seen from Supplementary Fig. 5c–f, the final errors (~20–25 μmol/kg) in an individual [CO_3_^2−^] reconstruction based on the Monte-Carlo are similar to those (~18–24 μmol/kg) based on quadratic addition of individual errors, justifying our major error estimation approach (i.e., quadratic addition).

### Subtropical western North Atlantic surface [CO_3_^2−^]

Because most of North Atlantic subtropical gyre waters circulate through the Caribbean Sea before being transported to the subpolar North Atlantic via the Gulf Stream, ODP 999 from Caribbean Sea is used to constrain past Gulf Stream carbonate chemistry^20^. To further test the feasibility of using ODP 999 to represent the first-order Gulf Stream [CO_3_^2−^] changes during the Holocene and LGM, we have estimated surface-water [CO_3_^2−^] for four sites from the wider subtropical western Atlantic region (latitude: 12–33°N, longitude: 61–91°W). Among these sites, KNR140–51GGC (33°N, 76°W) is located within the Gulf Stream today^71^. Because subtropical surface waters cycle multiple times through the upper ocean gyre circulations, it is possible that surface waters have been close to equilibrium with past atmospheric *p*CO_2_ (refs. ^21,22^). Therefore, we assume surface-water *p*CO_2_ of 270 and 194 ppm for the Holocene and LGM, respectively^72^. We assign a ±15 ppm error to surface-water *p*CO_2_ to account for any potential air–sea CO_2_ disequilibrium. For these sites, we use surface temperature and salinity reconstructions from previous publications^71,73–75^. ALK is calculated based on the same approach for ODP 999. The reconstructed in situ [CO_3_^2−^] values show some differences between cores, due to local T–S conditions. Since we are interested in air–sea CO_2_ exchange signals, we convert reconstructed in situ [CO_3_^2^^−^] into [CO_3_^2^^−^]_Norm_ using Eq. (11). As can be seen from Supplementary Fig. 6 and Supplementary Data 2, these cores show similar [CO_3_^2−^]_Norm_ values for the Holocene (~260 μmol/kg) and LGM (~300 μmol/kg) as ODP 999. Therefore, we argue that ODP 999 sufficiently records first-order Gulf Stream air–sea exchange carbonate chemistry for the Holocene and LGM. Because we aim to obtain a proxy-based estimates, we use ODP 999 data for calculations in the main text.

### Benthic B/Ca and δ^11^B to deep-water [CO_3_^2−^]

Most deep-water [CO_3_^2−^] values are reconstructed using benthic B/Ca (refs. ^25,47^) from [CO_3_^2−^]_downcore_ = [CO_3_^2−^]_PI_ + ΔB/Ca_downcore–coretop_/*k*, where [CO_3_^2−^]_PI_ is the preindustrial (PI) deep-water [CO_3_^2^^−^] value estimated from the GLODAP dataset^2^, ΔB/Ca_downcore–coretop_ represents the deviation of B/Ca of down-core samples from the core-top value, and *k* is the B/Ca–[CO_3_^2−^] sensitivity of *C. wuellerstorfi* (1.14 μmol/mol per μmol/kg) or *C. mundulus* (0.69 μmol/mol per μmol/kg)^25^. We use a reconstruction uncertainty of ±10 μmol/kg in [CO_3_^2−^] based on global core-top calibration samples^25,76^.

For cores BOFS 17 K and BOFS 11 K, new monospecific epifaunal benthic δ^11^B values were converted into deep-water [CO_3_^2−^] following the approach detailed in ref. ^77^. Briefly, benthic δ^11^B is assumed to directly reflect deep-water borate δ^11^B, as suggested by previous core-top calibration work^78^. Deep-water pH is calculated using benthic δ^11^B along with T_deep_ and S_deep_, similar to the approach to calculate surface-water pH at ODP 999 (refs. ^21,22^). We assume constant ALK at the studied sites (2313 μmol/kg at BOFS 17 K and 2310 μmol/kg at BOFS 11 K) in the past. Following ref. ^77^, a generous error of 100 μmol/kg is assigned to ALK estimates. We then calculate deep-water [CO_3_^2−^] from pH and ALK using the CO_2_sys program^28^. The integrated average uncertainty in deep-water [CO_3_^2−^] is ~±10 μmol/kg, based on quadratic addition of individual errors of ~±2 μmol/kg sourced from T_deep_ (±1 °C), ~±2 μmol/kg from S_deep_ (±1‰), ~±5 μmol/kg from ALK (±100 μmol/kg), and ~±8 μmol/kg from δ^11^B_borate_ (~±0.25‰). As demonstrated by Supplementary Fig. 5, the large ALK error only contributes a small uncertainty to the final [CO_3_^2−^] estimate. As shown in Fig. 3, benthic B/Ca and δ^11^B yield consistent deep-water [CO_3_^2^^−^] reconstructions for the Holocene and LGM.

### Benthic Cd/Ca to deep-water PO_4_

We follow the established approach^26,46,79^ to convert benthic (*C. wuellerstorfi*, *C. mundulus*, and *Uvigerina* spp.) foraminiferal Cd/Ca into deep-water Cd concentrations. Partition coefficients (*D*_Cd_) are used to calculate deep water Cd from: Cd (nmol/kg) = [(Cd/Ca)_foram_/*D*_Cd_] × 10. Bertram et al.^65^ used empirical *D*_Cd_ values of 2.3, 2.2, and 2.7 for BOFS 17, 14, and 11 K, respectively. However, these *D*_Cd_ values would result in Holocene Cd of 0.3–0.4 nmol/kg, higher than the observed value of ~0.25 nmol/kg from modern hydrographic measurements (Supplementary Fig. 7)^80^. This offset may suggest higher *D*_Cd_ values for the North Atlantic Ocean, which has been acknowledged recently^81^. We thus adjust D_Cd_ (~25% increase) so that the calculated Holocene deep-water Cd concentrations match modern measurements. This adjustment is supported by consistent Cd reconstructions from this study and previous reconstructions based on Cd/Ca measurements for *Hoeglundina elegans*. Compared to *Cibicidoides*, *D*_Cd_ into *H. elegans* is far less variable^79^. As can be seen from Supplementary Fig. 8, for cores with similar benthic δ^13^C from similar water depths (i.e., bathed in similar water masses), our Cd reconstructions match favorably with those based on *H. elegans* measurements^82^. Deep water Cd is converted into PO_4_ using the relationship based on the latest North Atlantic Ocean measurements (Supplementary Fig. 7)^80^. Using older published Cd–PO_4_ relationships^26,83^ only marginally affects our PO_4_ estimates.

Uncertainties associated with Cd and PO_4_ reconstructions are estimated as follows. Error for Cd is estimated using 2*σ*_Cd_ = $\sqrt{(2\sigma _{D_{\mathrm{Cd}}})^2 + (2\sigma _{{\mathrm{Cd}}/{\mathrm{Ca}}})^2}$, where $2\sigma _{D_{\mathrm{Cd}}}$ and $2\sigma _{\mathrm{Cd}/\mathrm{Ca}}$ (=5%) are errors for *D*_Cd_ and Cd/Ca, respectively. Due to poorly defined uncertainty for *D*_Cd_ from the literature, we assume an error of 50%, and then compare our final errors with literature estimates to assess the appropriateness of our calculations. Seawater PO_4_ is calculated from Cd using: PO_4_ = $\frac{{\mathrm{Cd} - b \pm (2\sigma _b)}}{{a \pm (2\sigma _a)}}$, where 2*σ*_*a*_ and 2*σ*_*b*_, respectively, represent 95% confidence errors associated with *a* and *b* (Supplementary Fig. 7b). The PO_4_ uncertainty was calculated from: $2\sigma _{{{\rm{PO}}_4}} = \sqrt {\left( {\partial _{{{\rm{PO}}_4}}{\mathrm{/}}\partial _a \cdot 2\sigma _a} \right)^2 + \left( {\partial _{{{\rm{PO}}_4}}{\mathrm{/}}\partial _b \cdot 2\sigma _b} \right)^2 + \left( {\partial _{{{\rm{PO}}_4}}/\partial _{\mathrm{Cd}} \cdot 2\sigma _{\mathrm{Cd}}} \right)^2}$, where $\partial _{{{\rm{PO}}_4}}/\partial _a$ = $\frac{{ - (\mathrm{Cd} - b)}}{{a^2}}$, $\partial _{{{\rm{PO}}_4}}/\partial _b$ = $\frac{{ - 1}}{a}$, and $\partial_{{{\rm{PO}}_4}}/\partial _{\mathrm{Cd}}$ = $\frac{1}{a}$. Our final errors on individual Cd and PO_4_ are ~0.12 nmol/kg (~55%) and ~0.5 μmol/kg (~50%), respectively. When compared with previously published uncertainties (~0.08 nmol/kg for Cd and ~0.17 μmol/kg for PO_4_)^46,68^, our error estimates are possibly too generous. Here we use ~50% error to be conservative. We encourage future work to improve uncertainty estimates for the benthic Cd/Ca proxy.

The oceanic residence time of PO_4_ is ~100,000 years^84^. The LGM deep ocean was possibly more reducing^85^, which might have facilitated sediment organic matter preservation, and, thus, PO_4_ removal from the ocean. However, this effect might have been compensated by decreased organic burial on continental slopes due to shallower LGM sea levels^86,87^. Considering the short (~10,000 years) last deglacial^84^, we assume that global PO_4_ and Cd reservoirs remained constant between the Holocene and LGM. Our reconstructions (Fig. 3) are consistent with high benthic δ^13^C and low benthic Cd/Ca at numerous glacial North Atlantic mid-depth sites^23,31,46,65,88,89^.

### Deep-water temperature and salinity estimates

Deep-water temperature (T_deep_) is estimated from the ice volume corrected benthic δ^18^O (δ^18^O_IVC_) and the δ^18^O-temperature equation of Marchitto et al.^90^ from *T*_deep_ = 2.5 − (δ^18^O_IVC_ − 2.8)/0.224, where δ^18^O_IVC_ = δ^18^O_benthic_ − δ_18_O_global_sealevel_. δ^18^O_global_sealevel_ was estimated from sea level curves^86,87^ with a global δ^18^O_seawater_−sea level scaling of 0.0085‰/m (ref. ^91^). Deep-water salinity (S_deep_) is calculated by: *S*_deep_ = *S*_core_top_ + 1.11 × δ^18^O_global_sealevel_, where S_core_top_ is the modern S_deep_ (35.06, 34.926, and 34.893 at BOFS 17 , 11 , and 14 K, respectively^2^) and the term 1.11 is the scaling term for a global S−δ^18^O_global_sealevel_ relationship^29,91^. We assume ±1 °C and ±1‰ uncertainties in T_deep_ and S_deep_, respectively. Use of other methods to estimate T_deep_ and S_deep_ negligibly affects our conclusions, due to relatively weak sensitivities of [CO_3_^2−^]_Norm_ to T and S changes (Fig. 4).

### Uncertainties and statistical analyses

Uncertainties associated with [CO_3_^2−^] and PO_4_ were evaluated using a Monte-Carlo approach^92,93^. Errors associated with the chronology (*x*-axis) and [CO_3_^2−^] and PO_4_ reconstructions (*y*-axis) are considered during error propagation. Age errors are assumed to be ±3000 years for the three BOFS cores. Methods to calculate errors associated with individual [CO_3_^2−^] and PO_4_ reconstructions (*y*-axis) are given above. All data points were sampled separately and randomly 5000 times within their chronological and [CO_3_^2−^] or PO_4_ uncertainties and each iteration was then interpolated linearly. At each time step, the probability maximum and data distribution uncertainties of the 5000 iterations were assessed. Figure 3 shows probability maxima (bold curves) and ±95% (light gray; 2.5−97.5th percentile) probability intervals for the data distributions, including chronological and proxy uncertainties. For details, see refs. ^92,93^.

For a time period (e.g., Holocene) where multiple analyses are available, uncertainties are calculated following the method from ref. ^94^ by 2*σ* = $\sqrt {[\mathop {\sum }\nolimits_{i = 1}^n (2\sigma _i)^2]/n}$, where *n* is the number of reconstructions and 2*σ*_*i*_ is the error associated with individual reconstruction. For [CO_3_^2−^] or [CO_3_^2−^]_Norm_ offsets between the Holocene and LGM, 2*σ* = $\sqrt {(2\sigma _{\mathrm{Holocene}})^2 + (2\sigma _{\mathrm{LGM}})^2}$, where $2{\sigma} _{\mathrm{Holocene}}$ and $2{\sigma} _{\mathrm{LGM}}$ are 2*σ* of Holocene and LGM values, respectively. Other methods (e.g., weighted mean)^95^ would give similar results.

When using Eq. (11) to calculate [CO_3_^2−^]_Norm_, errors from various sensitivities are <1.5 μmol/kg (see Supplementary Data 8 for crosschecking). Because [CO_3_^2^^−^] is normalized to a constant condition (i.e., no error with final T–S–P), the error in [CO_3_^2−^]_Norm_ is largely sourced from [CO_3_^2−^] reconstruction uncertainties. For surface water [CO_3_^2−^]_Norm_ calculations, T and S errors are already included in surface [CO_3_^2^^−^] reconstructions. For calculations associated with deep waters, [CO_3_^2−^]_Norm_ errors are ~0.5, ~3.5, and ~0.1 μmol/kg from ± 1 °C in T, ±1‰ in S, and ±50 dbar in P, respectively. Therefore, these uncertainties (already included in error calculations) are relatively less important compared to the reconstruction error of ±10 μmol/kg for deep water [CO_3_^2−^].

## Supplementary information

Supplementary Information

Peer Review File

Description of Additional Supplementary Files

Supplementary Data 1

Supplementary Data 2

Supplementary Data 3

Supplementary Data 4

Supplementary Data 5

Supplementary Data 6

Supplementary Data 7

Supplementary Data 8

Supplementary Data 9

## Data Availability

The data reported in the paper are presented in Supplementary Data.

## References

[1] Takahashi T (2009). Climatological mean and decadal change in surface ocean pCO2, and net sea-air CO2 flux over the global oceans. Deep Sea Res Part II.

[2] Key R. M., Kozyr A., Sabine C. L., Lee K., Wanninkhof R., Bullister J. L., Feely R. A., Millero F. J., Mordy C., Peng T.-H. (2004). A global ocean carbon climatology: Results from Global Data Analysis Project (GLODAP). Global Biogeochemical Cycles.

[3] Broecker W, Peng TH (1992). Interhemispheric transport of carbon dioxide by ocean circulation. Nature.

[4] Gloor M (2003). A first estimate of present and preindustrial air-sea CO2 flux patterns based on ocean interior carbon measurements and models. Geophys. Res. Lett..

[5] Ciais P (2012). Large inert carbon pool in the terrestrial biosphere during the Last Glacial Maximum. Nat. Geosci..

[6] Sigman DM, Hain MP, Haug GH (2010). The polar ocean and glacial cycles in atmospheric CO2 concentration. Nature.

[7] Yu J (2010). Loss of carbon from the deep sea since the Last Glacial Maximum. Science.

[8] Martinez-Garcia A (2014). Iron fertilization of the Subantarctic Ocean during the last ice age. Science.

[9] Anderson RF (2009). Wind-driven upwelling in the Southern Ocean and the deglacial rise in atmospheric CO2. Science.

[10] Anderson LA, Sarmiento JL (1994). Redfield ratios of remineralization determined by nutrient data-analysis. Glob. Biogeochem. Cycle.

[11] Keeling CD, Heimann M (1986). Meridional eddy diffusion model of the transport of atmospheric carbon dioxide: 2. Mean annual carbon cycle. J. Geophys. Res..

[12] Toggweiler, J. R., Murnane, R., Carson, S., Gnanadesikan, A. & Sarmiento, J. L. Representation of the carbon cycle in box models and GCMs: 2. Organic pump. *Glob. Biogeochem. Cycle* **17**, 1027 (2003).

[13] Toggweiler, J. R., Gnanadesikan, A., Carson, S., Murnane, R. & Sarmiento, J. L. Representation of the carbon cycle in box models and GCMs: 1. Solubility pump. *Glob. Biogeochem. Cycle* **17**, 1026 (2003).

[14] Palter JB, Lozier MS, Barber RT (2005). The effect of advection on the nutrient reservoir in the North Atlantic subtropical gyre. Nature.

[15] Hain, M. P., Sigman, D. M. & Haug, G. H. The Biological Pump in the Past. In *Treatise on Geochemistry* 2nd ed. 485–517 (Elsevier, 2013).

[16] Zeebe RE, Wolf-Gladrow DA (2001). CO2 in Seawater: Equilibrium, Kinetics, Isotopes.

[17] Talley LD (2013). Closure of the global overturning circulation through the Indian, Pacific, and Southern Oceans: schematics and transports. Oceanography.

[18] Lozier MS (2010). Deconstructing the conveyor belt. Science.

[19] Foukal, N. P. & Lozier, M. S. No inter-gyre pathway for sea-surface temperature anomalies in the North Atlantic. *Nat. Commun*. **7**, 11333 (2016). 10.1038/ncomms11333 PMC4844697 27103496

[20] Johns WE, Townsend TL, Fratantoni DM, Wilson WD (2002). On the Atlantic inflow to the Caribbean Sea. Deep Sea Res. Part I: Oceanogr. Res. Pap..

[21] Foster GL (2008). Seawater pH, pCO2 and [CO32-] variations in the Caribbean Sea over the last 130 kyr; a boron isotope and B/Ca study of planktic foraminifera. Earth Planet Sci. Lett..

[22] Henehan MJ (2013). Calibration of the boron isotope proxy in the planktonic foraminifera Globigerinoides ruber for use in palaeo-CO2 reconstruction. Earth Planet Sci. Lett..

[23] Curry WB, Oppo D (2005). Glacial water mass geometry and the distribution of d13C of SCO2 in the western Altantic Ocean. Paleoceanography.

[24] Yu JM, Elderfield H, Piotrowski A (2008). Seawater carbonate ion-d13C systematics and application to glacial-interglacial North Atlantic ocean circulation. Earth Planet Sci. Lett..

[25] Yu JM, Elderfield H (2007). Benthic foraminiferal B/Ca ratios reflect deep water carbonate saturation state. Earth Planet Sci. Lett..

[26] Boyle EA (1988). Cadmium: Chemical tracer of deepwater paleoceanography. Paleoceanography.

[27] Boyle EA (1992). Cadmium and d13C paleochemical ocean distributions during the stage-2 glacial maximum. Annu Rev. Earth Planet Sci..

[28] Pelletier, G., Lewis, E. & Wallace, D. *A Calculator for the CO2 System in Seawater for Microsoft Excel/VBA*, 1.0 ed. (Washington State Department of Ecology, Olympia; Brookhaven National Laboratory, Upton, 2005).

[29] Sarmiento, J. L. & Gruber, N. *Ocean Biogeochemical Dynamics*. (Princeton University Press, Princeton, 2006).

[30] Takahashi T, Broecker W, Langer S (1985). Redfield ratio based on chemical data from isopycnal surfaces. J. Geophys. Res..

[31] Lynch-Stieglitz J (2007). Atlantic meridional overturning circulation during the Last Glacial Maximum. Science.

[32] Lynch-Stieglitz J, van Geen A, Fairbanks RG (1996). Interocean exchange of Glacial North Atlantic intermediate water: evidence from Subantarctic Cd/Ca and carbon isotope measurements. Paleoceanography.

[33] Makou, M. C., Oppo, D. W. & Curry, W. B. South Atlantic intermediate water mass geometry for the last glacial maximum from foraminiferal Cd/Ca. *Paleoceanography* **25**, PA4101 (2010).

[34] McManus JF, Francois R, Gherardi JM, Keigwin LD, Brown-Leger S (2004). Collapse and rapid resumption of Atlantic meridional circulation linked to deglacial climate changes. Nature.

[35] Howe, J. N. W. et al. North Atlantic deep water production during the Last Glacial Maximum. *Nat. Commun*. **7** 11765 (2016). 10.1038/ncomms11765 PMC4895795 27256826

[36] Gebbie G (2014). How much did Glacial North AtlanticWater shoal?. Paleoceanography.

[37] Keigwin, L. & Swift, S. A. Carbon isotope evidence for a northern source of deep water in the glacial western North Atlantic. *Proc. Natl Acad. Sci. USA* **114**, 2831–2835 (2017). 10.1073/pnas.1614693114 PMC5358345 28193884

[38] Johnson, G. C. Quantifying Antarctic bottom water and north atlantic deep water volumes. *J. Geophys. Res. Oceans* **113**, C05027 (2008).

[39] Broecker W (1998). How much deep water is formed in the Southern Ocean?. J. Geophys. Reasearch.

[40] Yu J, Thornalley DJR, Rae J, McCave IN (2013). Calibration and application of B/Ca, Cd/Ca, and d11B in Neogloboquadrina pachyderma (sinistral) to constrain CO2 uptake in the subpolar North Atlantic during the last deglaciation. Paleoceanography.

[41] Martinez-Boti MA (2015). Boron isotope evidence for oceanic carbon dioxide leakage during the last deglaciation. Nature.

[42] Gray WR (2018). Deglacial upwelling, productivity and CO2 outgassing in the North Pacific Ocean. Nat. Geosci..

[43] Liu Z (2009). Transient simulation of last deglaciation with a new mechanism for Bolling-Allerod warming. Science.

[44] Waelbroeck C (2009). Constraints on the magnitude and patterns of ocean cooling at the Last Glacial Maximum. Nat. Geosci..

[45] Straub M (2013). Nutrient conditions in the subpolar North Atlantic during the last glacial period reconstructed from foraminifera-bound nitrogen isotopes. Paleoceanography.

[46] Marchitto, T. & Broecker, W. Deep water mass geometry in the glacial Atlantic Ocean: a review of constraints from the paleonutrient proxy Cd/Ca. *Geochem. Geophys. Geosyst.* **7**, Q12003 (2006).

[47] Yu J (2016). Sequestration of carbon in the deep Atlantic during the last glaciation. Nat. Geosci..

[48] Hoogakker BAA, Elderfield H, Schmiedl G, McCave IN, Rickaby REM (2015). Glacial-interglacial changes in bottom-water oxygen content on the Portuguese margin. Nat. Geosci..

[49] Anderson Robert F., Sachs Julian P., Fleisher Martin Q., Allen Katherine A., Yu Jimin, Koutavas Athanasios, Jaccard Samuel L. (2019). Deep‐Sea Oxygen Depletion and Ocean Carbon Sequestration During the Last Ice Age. Global Biogeochemical Cycles.

[50] Chen TY (2015). Synchronous centennial abrupt events in the ocean and atmosphere during the last deglaciation. Science.

[51] Jaccard SL, Galbraith ED, Martinez-Garcia A, Anderson RF (2016). Covariation of deep Southern Ocean oxygenation and atmospheric CO2 through the last ice age. Nature.

[52] Crichton KA, Bouttes N, Roche DM, Chappellaz J, Krinner G (2016). Permafrost carbon as a missing link to explain CO2 changes during the last deglaciation. Nat. Geosci..

[53] Ferrari R (2014). Antarctic sea ice control on ocean circulation in present and glacial climates. Proc. Natl Acad. Sci. USA.

[54] Hain, M. P., Sigman, D. M. & Haug, G. H. Carbon dioxide effects of Antarctic stratification, North Atlantic Intermediate Water formation, and subantarctic nutrient drawdown during the last ice age: diagnosis and synthesis in a geochemical box model. *Glob. Biogeochem. Cycle* **24**, GB4023 (2010).

[55] Mehrbach C, Culberso CH, Hawley JE, Pytkowic RM (1973). Measurement of apparent dissociation-constants of carbonic-acid in seawater at atmospheric-pressure. Limnol. Oceanogr..

[56] Dickson AG (1990). Thermodynamics of the dissociation of boric-acid in synthetic seawater from 273.15K to 318.15K. Deep Sea Res. Part A.

[57] Lee K (2010). The universal ratio of boron to chlorinity for the North Pacific and North Atlantic oceans. Geochim. Cosmochim. Acta.

[58] Menviel L., Yu J., Joos F., Mouchet A., Meissner K. J., England M. H. (2017). Poorly ventilated deep ocean at the Last Glacial Maximum inferred from carbon isotopes: A data-model comparison study. Paleoceanography.

[59] Schmidt MW, Spero HJ, Lea DW (2004). Links between salinity variation in the Caribbean and North Atlantic thermohaline circulation. Nature.

[60] Rosenthal Y, Boyle EA, Slowey N (1997). Temperature control on the incorporation of magnesium, strontium, fluorine, and cadmium into benthic foraminiferal shells from Little Bahama Bank: prospects for thermocline paleoceanography. Geochim. Cosmochim. Acta.

[61] Barker, S., Greaves, M. & Elderfield, H. A study of cleaning procedures used for foraminiferal Mg/Ca paleothermometry. *Geochem. Geophys. Geosyst*. **4**, 8407 (2003).

[62] Yu, J., Elderfield, H., Greaves, M. & Day, J. Preferential dissolution of benthic foraminiferal calcite during laboratory reductive cleaning. *Geochem. Geophys. Geosyst.* **8**, Q06016 (2007).

[63] Manighetti B, McCave IN, Maslin M, Shackleton NJ (1995). Chronology for climate change: developing age models for the Biogeochemical Ocean Flux Study cores. Paleoceanography.

[64] Barker, S., Kiefer, T. & Elderfield, H. Temporal changes in North Atlantic circulation constrained by planktonic foraminiferal shell weights. *Paleoceanography* **19**, PA3008 (2004).

[65] Bertram CJ, Elderfield H, Shackleton NJ, Macdonald JA (1995). Cadmium/calcium and carbon-isotope reconstructions of the glacial northeast Atlantic Ocean. Paleoceanography.

[66] Yu JM, Elderfield H (2008). Mg/Ca in the benthic foraminifera *Cibicidoides wuellerstorfi* and *Cibicidoides mundulus*: temperature versus carbonate ion saturation. Earth Planet Sci. Lett..

[67] Yu JM, Day J, Greaves M, Elderfield H (2005). Determination of multiple element/calcium ratios in foraminiferal calcite by quadrupole ICP-MS. Geochem Geophys Geosyst.

[68] Lear CH (2016). Breathing more deeply: deep ocean carbon storage during the mid-Pleistocene climate transition. Geology.

[69] Boyle E, Keigwin LD (1985). Comparison of Atlantic and Pacific paleochemical records for the last 215,000 years: changes in deep ocean circulation and chemical inventories. Earth Planet Sci. Lett..

[70] Foster GL, Sexton PF (2014). Enhanced carbon dioxide outgassing from the eastern equatorial Atlantic during the last glacial. Geology.

[71] Carlson AE (2008). Subtropical Atlantic salinity variability and Atlantic meridional circulation during the last deglaciation. Geology.

[72] Lüthi D (2008). High-resolution carbon dioxide concentration record 650,000–800,000 years before present. Nature.

[73] Ziegler M, Nürnberg D, Karas C, Tiedemann R, Lourens LJ (2008). Persistent summer expansion of the Atlantic Warm Pool during glacial abrupt cold events. Nat. Geosci..

[74] Flower BP, Hastings DW, Hill HW, Quinn TM (2004). Phasing of deglacial warming and Laurentide Ice Sheet meltwater in the Gulf of Mexico. Geology.

[75] Rühlemann C, Mulitza S, Müller PJ, Wefer G, Zahn R (1999). Warming of the tropical Atlantic Ocean and slowdown of thermohaline circulation during the last deglaciation. Nature.

[76] Yu J (2013). Responses of the deep ocean carbonate system to carbon reorganization during the Last Glacial–interglacial cycle. Quat. Sci. Rev..

[77] Yu J, Foster GL, Elderfield H, Broecker WS, Clark E (2010). An evaluation of benthic foraminiferal B/Ca and d11B for deep ocean carbonate ion and pH reconstructions. Earth Planet Sci. Lett..

[78] Rae JWB, Foster GL, Schmidt DN, Elliott T (2011). Boron isotopes and B/Ca in benthic foraminifera: proxies for the deep ocean carbonate system. Earth Planet Sci. Lett..

[79] Boyle EA, Labeyrie L, Duplessy JC (1995). Calcitic foraminiferal data confirmed by cadmium in aragonitic Hoeglundina—application to the Last Glacial Maximum in the northern Indian Ocean. Paleoceanography.

[80] Schlitzer R (2018). The GEOTRACES intermediate data product 2017. Chem. Geol..

[81] Oppo D (2018). Data constraints on Glacial Atlantic water mass geometry and properties. Paleoceanogr. Paleoclimatol..

[82] Came RE, Oppo DW, Curry WB, Lynch-Stieglitz J (2008). Deglacial variability in the surface return flow of the Atlantic meridional overturning circulation. Paleoceanography.

[83] Elderfield H, Rickaby REM (2000). Oceanic Cd/P ratio and nutrient utilization in the glacial Southern Ocean. Nature.

[84] Delaney ML, Boyle EA (1987). Cd/Ca in late miocene benthic foraminifera and changes in the global organic-carbon budget. Nature.

[85] Jaccard SL, Galbraith ED (2012). Large climate-driven changes of oceanic oxygen concentrations during the last deglaciation. Nat. Geosci..

[86] Stanford, J. D. et al. Timing of meltwater pulse 1a and climate responses to meltwater injections. *Paleoceanography* **21**, PA4103 (2006).

[87] Lambeck K, Rouby H, Purcell A, Sun YY, Sambridge M (2014). Sea level and global ice volumes from the Last Glacial Maximum to the Holocene. P Natl. Acad. Sci. USA.

[88] Oppo DW, Lehman SJ (1993). Mid-depth circulation of the subpolar North Atlantic during the last glacial maximum. Science.

[89] Rickaby REM, Elderfield H (2005). Evidence from the high-latitude North Atlantic for variations in Antarctic Intermediate water flow during the last deglaciation. Geochem. Geophys. Geosyst..

[90] Marchitto TM (2014). Improved oxygen isotope temperature calibrations for cosmopolitan benthic foraminifera. Geochim. Cosmochim. Acta.

[91] Waelbroeck C (2002). Sea-level and deep water temperature changes derived from benthic foraminifera isotopic records. Quat. Sci. Rev..

[92] Grant KM (2012). Rapid coupling between ice volume and polar temperature over the past 150,000 years. Nature.

[93] Rohling EJ (2014). Sea-level and deep-sea-temperature variability over the past 5.3 million years. Nature.

[94] Harris, D. C. *Quantitative Chemical Analysis*, 6th ed. (W. H. Freeman and Company, New York, 2002).

[95] Taylor, J. R. *An Introduction to Error Analysis*. (University Science Books, Sausalito, California, 1982).

[96] Schlitzer, R. *Ocean Data View*. http://odv.awi-bremerhaven.de (2006).

