# Supplementary material for: More efficient North Atlantic carbon pump during the Last Glacial Maximum
Source: Nat Commun. 2019 May 15;10:2170. doi: 10.1038/s41467-019-10028-z (PMC6520411; doi:10.1038/s41467-019-10028-z)
Supplement: Supplementary file 1 — Supplementary Information [file 41467_2019_10028_MOESM1_ESM.pdf]

Supplementary Information for

More efficient North Atlantic carbon pump during the Last Glacial Maximum

Yu et al.

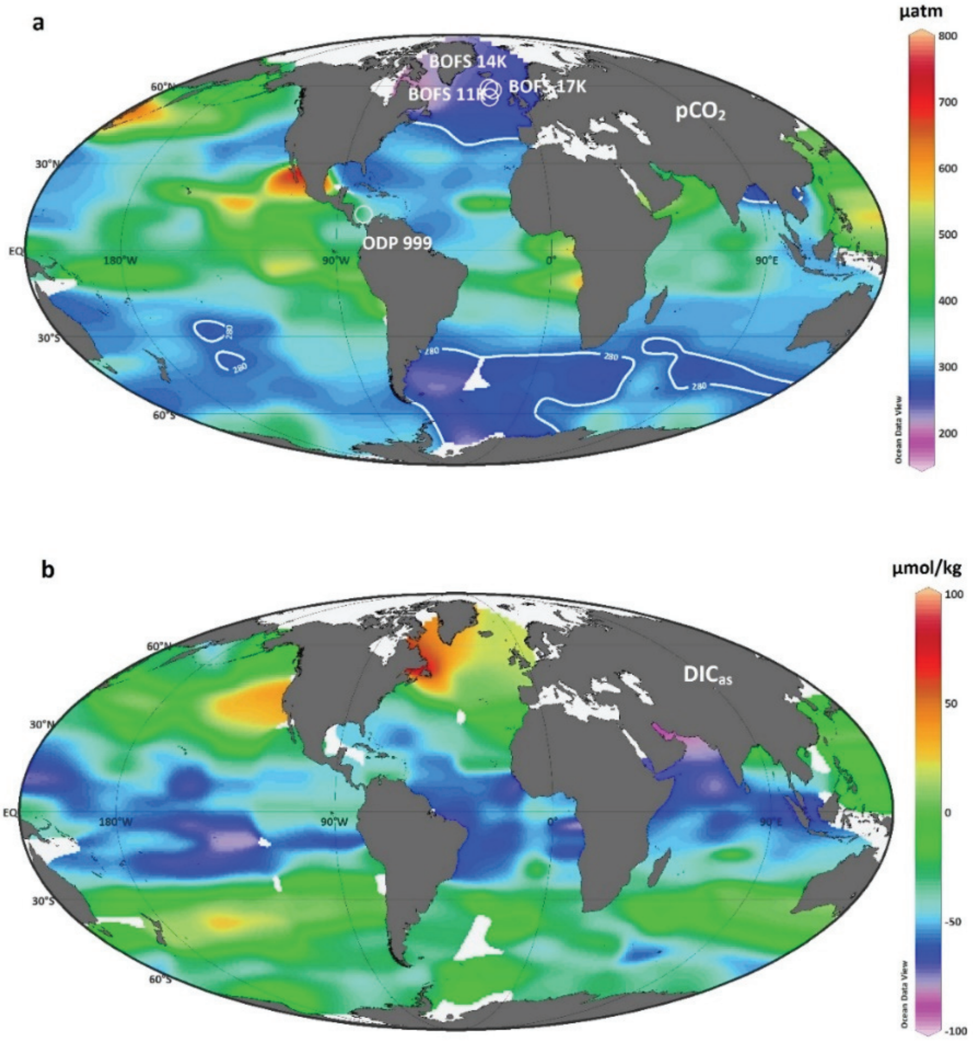

Supplementary Figure 1. Preindustrial (a) surface-water pCO$_2$ and (b) DIC$_{as}$. In **a**, white curves indicate the pCO$_2$ = 280 µatm value and white circles denote locations of the studied cores. In the North Atlantic Ocean, surface water pCO$_2$ is lower than the contemporary atmospheric pCO$_2$ of 280 µatm. This causes CO$_2$ invasion into the ocean, which is reflected by positive DIC$_{as}$ (**b**). DIC$_{as}$ is calculated using Equation (1) with C/PO$_4$ = 127. Maps are generated based on the GLODAP dataset[7] using Ocean Data View[8].

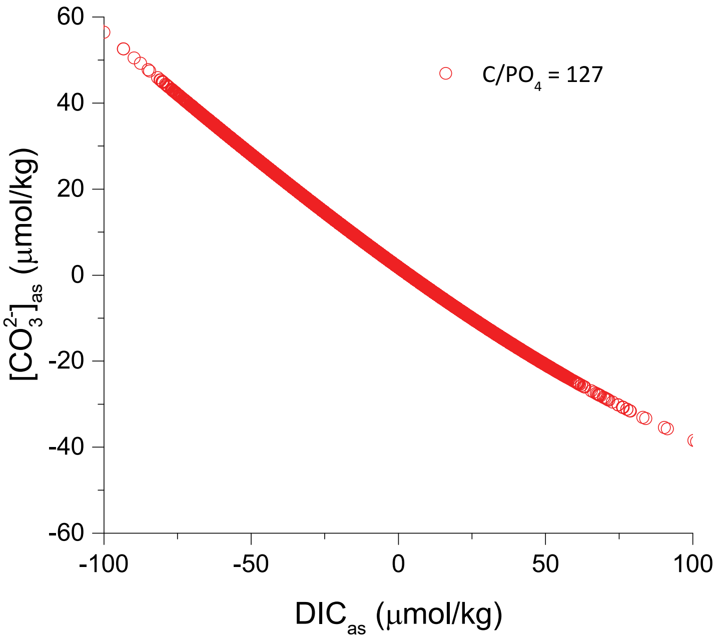

Supplementary Figure 2. Preindustrial DIC_{as} vs. $[CO_3^{2-}]_{as}$. Data are from 75°W to 15°E from the GLODAP dataset (n = 14,078)[7]. The $[CO_3^{2-}]_{as}/DIC_{as}$ slope is ~-0.6. The tight correlation suggests that $[CO_3^{2-}]_{as}$ changes reflect those in DIC_{as}, and hence reflect air-sea CO_2 exchange. See Methods for calculation methods.

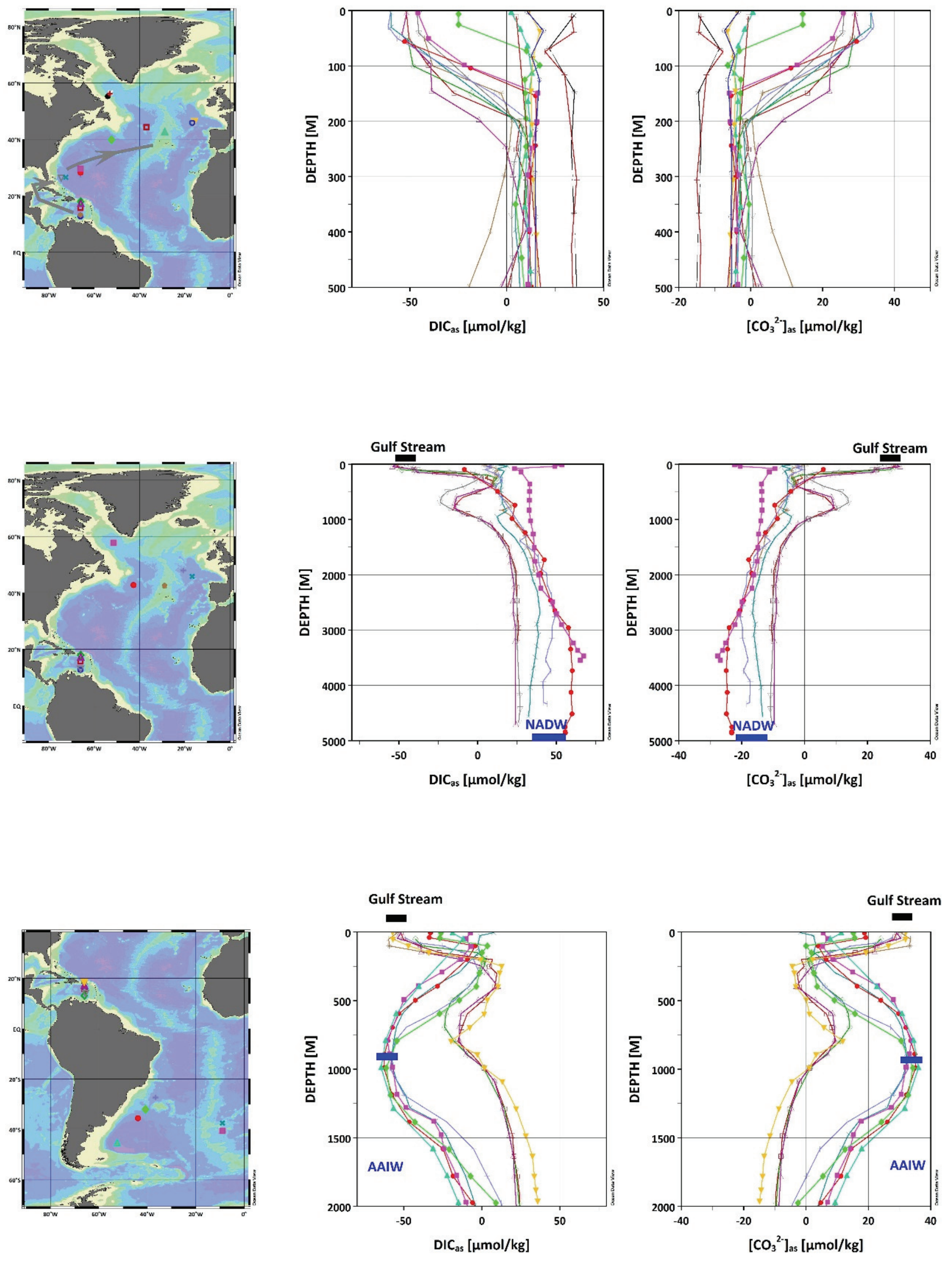

Supplementary Figure 3. Bathymetric distributions of [CO_3^{2-}]$_{as}$ and DIC$_{as}$ in the preindustrial Atlantic. Top row: changes along the Gulf Stream pathway. [CO_3^{2-}]$_{as}$ and DIC$_{as}$ only undergo minor changes within the Caribbean Sea; major changes start from >~35ºN where substantial atmospheric CO_2 absorption occurs. **Middle row:** Surface Gulf Stream signatures compared with NADW. **Bottom row**: Gulf Stream and AAIW have similar [CO_3^{2-}]$_{as}$ and DIC$_{as}$ signatures.

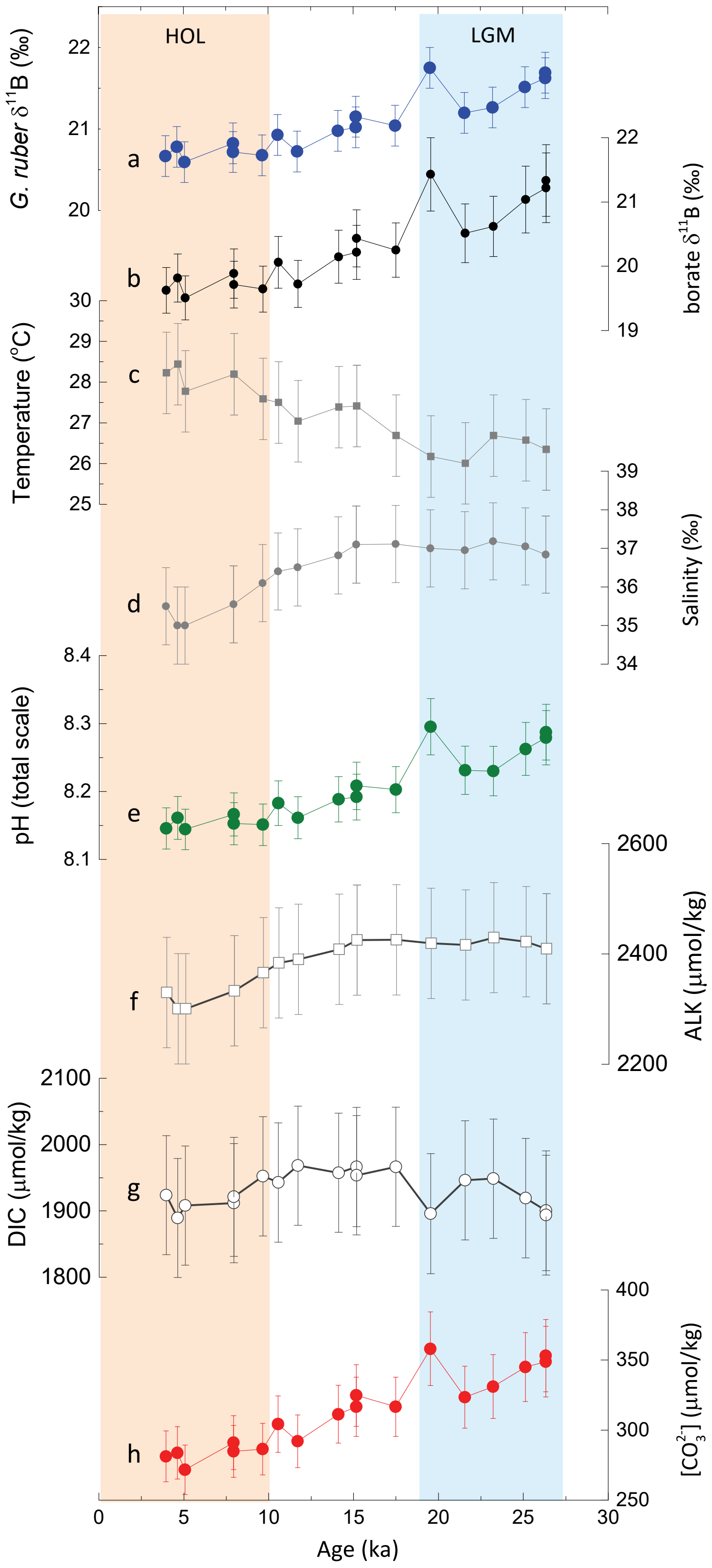

Supplementary Figure 4. ODP Site 999 surface-water reconstructions. From top to bottom, it shows *G. ruber* (w) $\delta^{11}B$ (ref. [9]) (**a**) and reconstructed surface-water borate $\delta^{11}B$ (**b**), temperature[10] (**c**), salinity[10] (**d**), pH (**e**), ALK (**f**), DIC (**g**), and $[CO_3^{2-}]$ (**h**). Error bars: ±2σ.

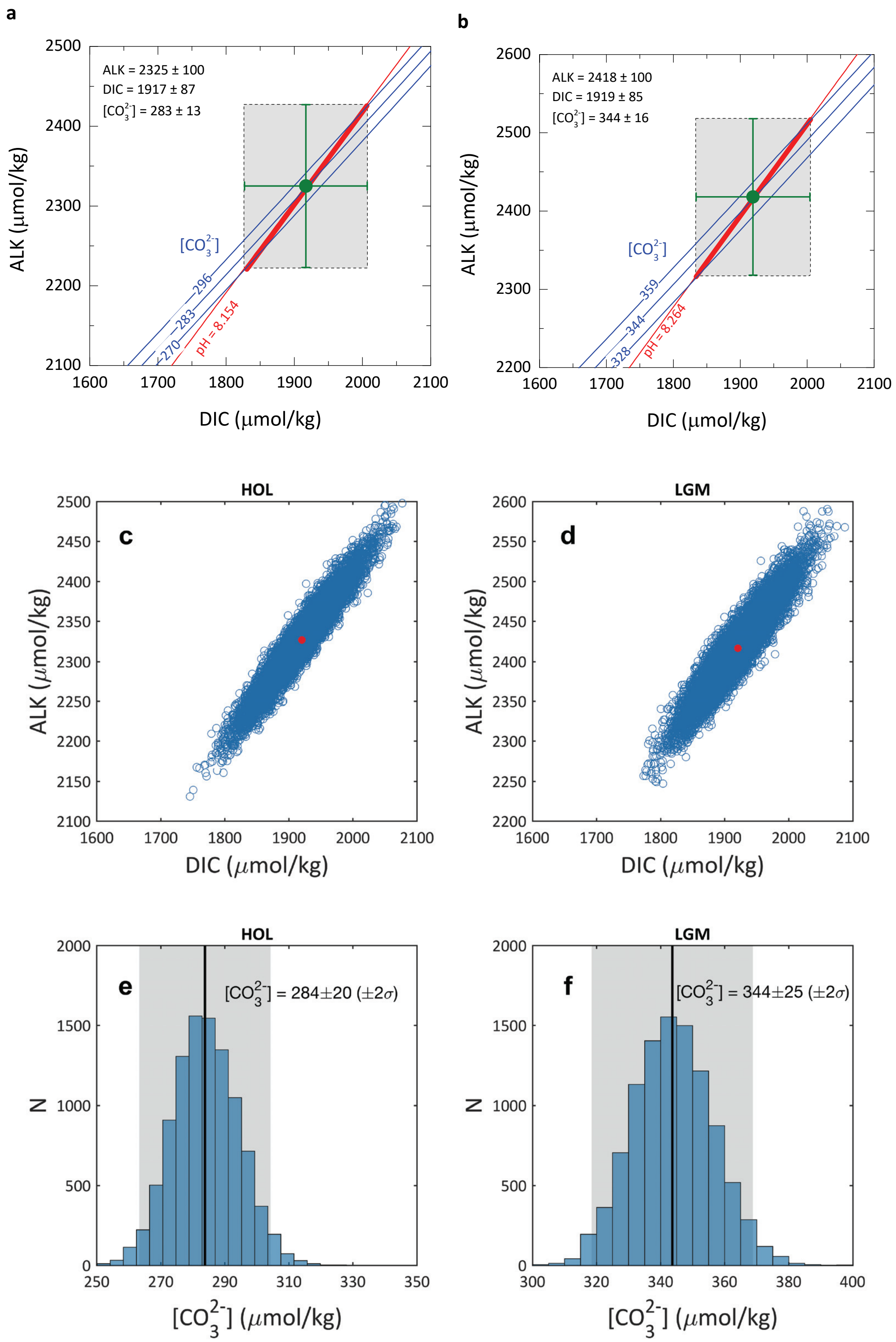

Supplementary Figure 5. Seawater [CO_3^{2-}] estimates for ODP 999. (**a**, **c**, **e**), Holocene mean. (**b**, **d, f**), LGM mean. In (**a**, **b**), for clarity, we only show [CO_3^{2-}] errors (±2σ.) sourced from ALK errors. Given the constraint from pH, ALK and DIC must change along the bold red lines. The shaded areas, covered by the ALK and DIC uncertainties (green error bars), are ***<u>not</u>*** possible. In (**c-f**), all errors from $T_{surface}$, $S_{surface}$, $ALK_{surface}$, and $pH_{surface}$ are included using a Monte Carlo approach[11]. Red dots in (**c**, **d**) show mean values. The finally reported [CO_3^{2-}] reconstructions include errors from all individual sources (see Supplementary Data 1).

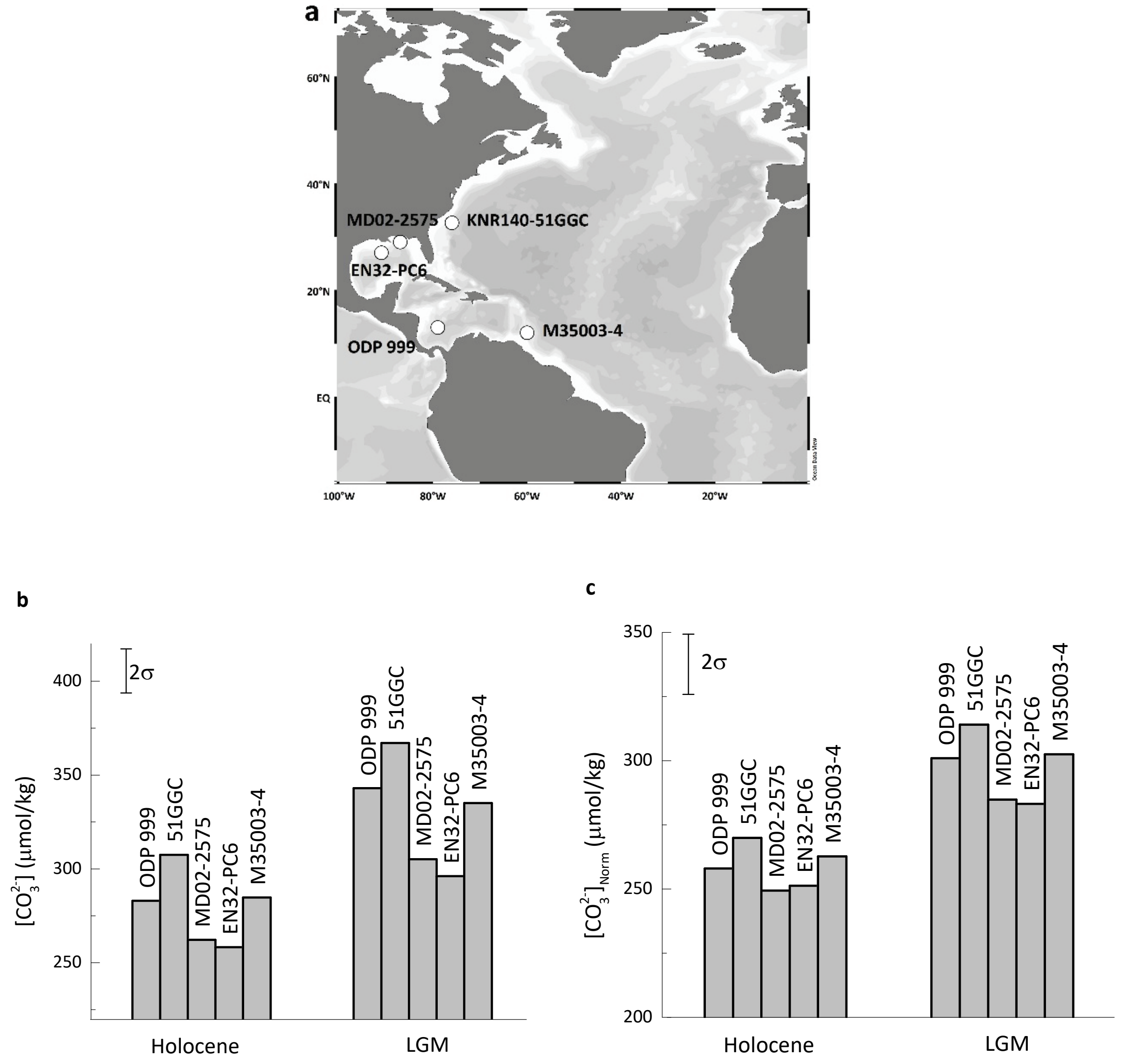

Supplementary Figure 6. Similar [CO_3^{2-}]$_{Norm}$ for ODP 999 and other sites from the broader subtropical western North Atlantic. a, Map showing core locations. **b**, [CO_3^{2-}] for the Holocene and LGM. **c**, as **b** but for [CO_3^{2-}]$_{Norm}$. [CO_3^{2-}]$_{Norm}$ for ODP 999 is calculated using $\delta^{11}B$ derived pH and ALK, while for other cores [CO_3^{2-}]$_{Norm}$ is based on the assumption of surface-atmosphere pCO_2 equilibrium within ±15 ppm.

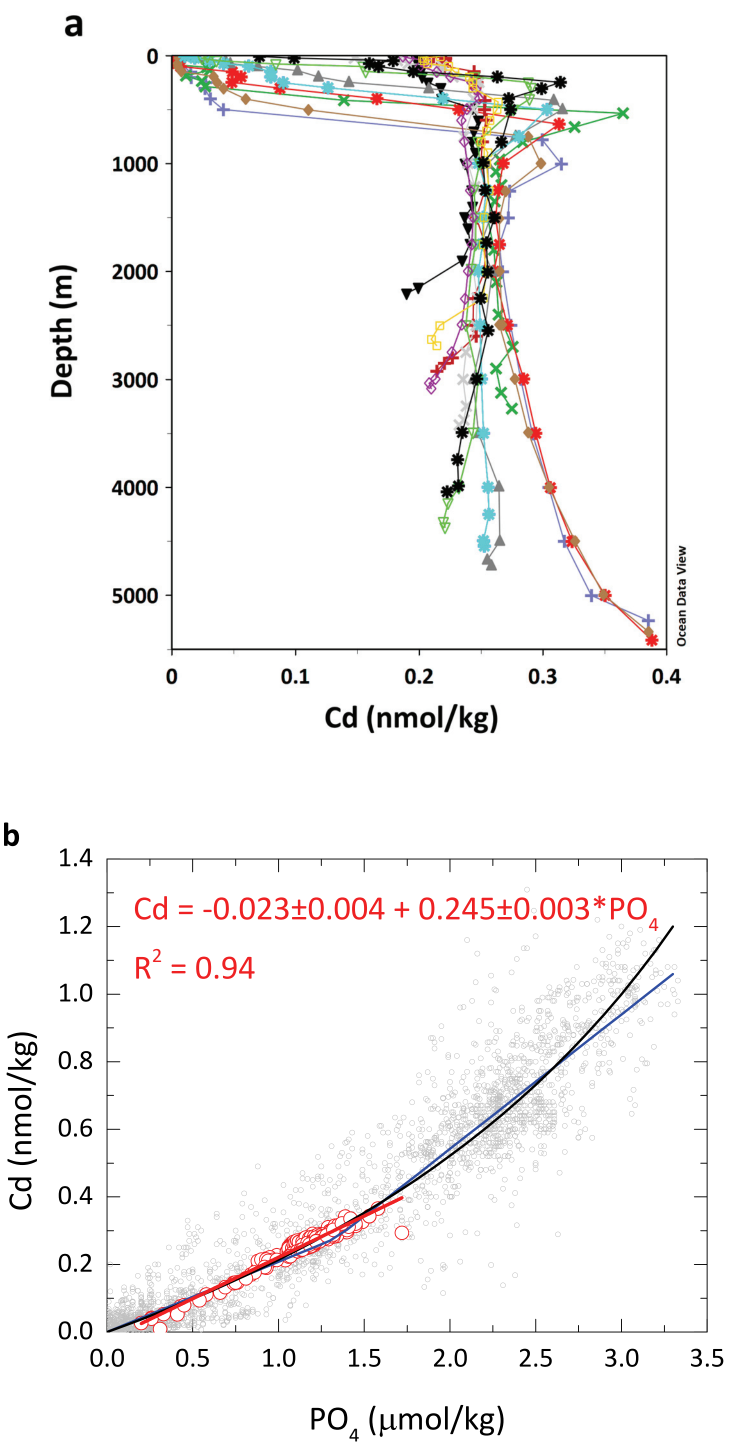

Supplementary Figure 7. Seawater Cd and PO_4 from the North Atlantic. a, Bathymetric Cd profiles for the North Atlantic[12]. **b**, Correlations between seawater Cd and PO_4. The red line is the best fit for the latest Cd-PO_4 data[12] (red circles; n = 307, >500 m water depth) from the North Atlantic (>30°N). Small grey circles are compiled literature data from refs [13-15,16 and references therein]. Blue and black curves represent correlations from Boyle[17] and Elderfield & Rickaby[13], respectively. Errors are ±2σ.

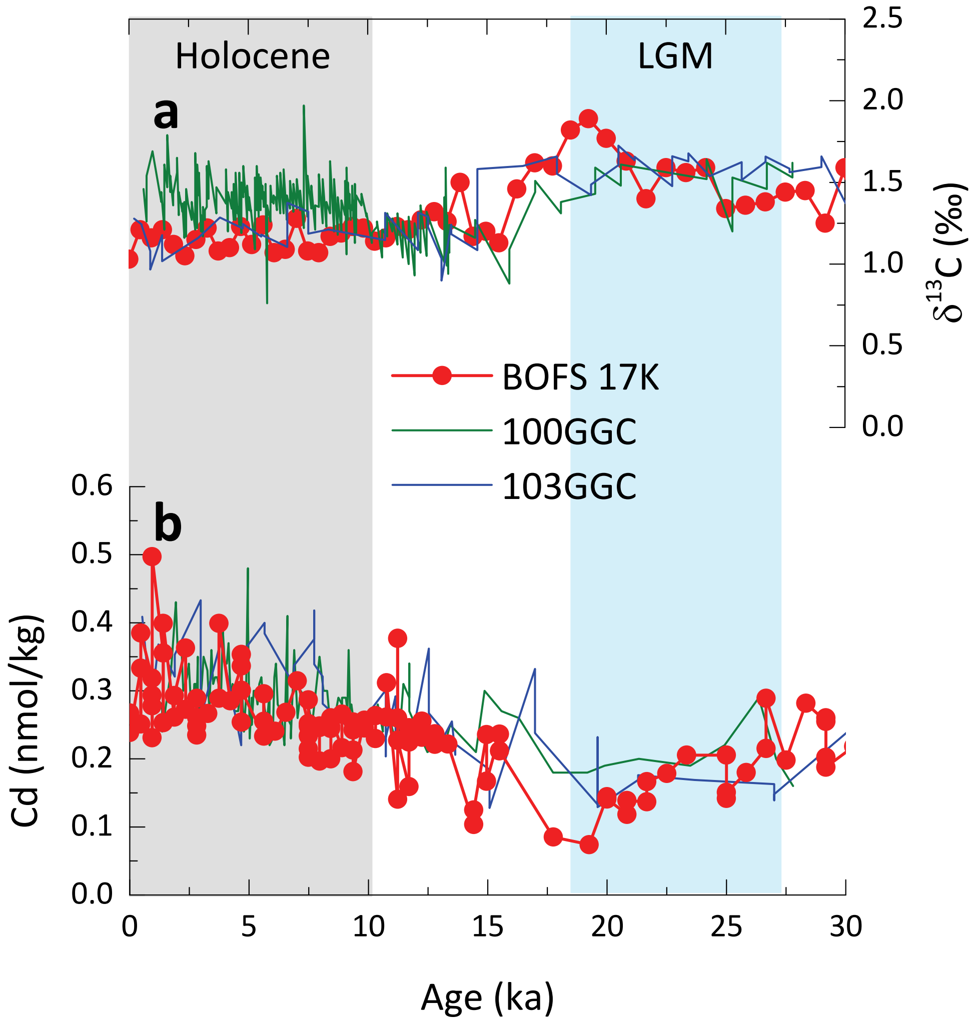

Supplementary Figure 8. Comparison of Cd from BOFS 17K and other cores. a, Benthic $\delta^{13}C$. Similar $\delta^{13}C$ values suggest that cores were possibly bathed in the same water mass, namely GNAIW, during the LGM. **b**, Deep water Cd reconstructed using *Cibicidoides* from BOFS 17K (58°N, 16.5°W, 1150 m), and *H. elegans* from 100GGC (26°N, 78°W, 1057 m) and 103GGC (26°N, 78°W, 965 m)[18]. The three cores are from similar water depths with comparable benthic $\delta^{13}C$. Similar Cd from three cores supports the validity of BOS 17K Cd reconstructions.

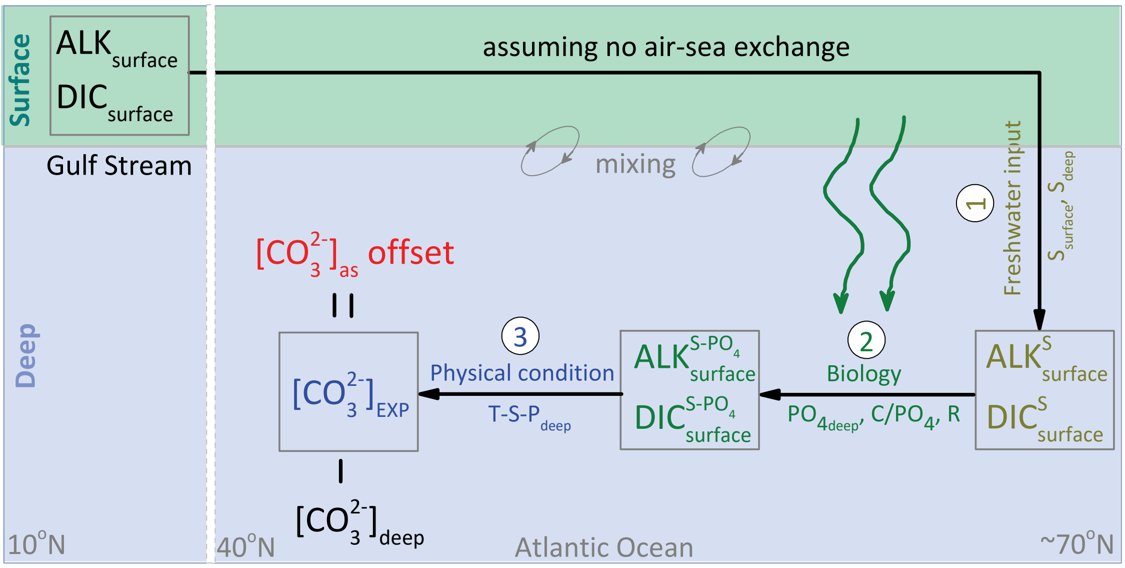

Supplementary Figure 9. Procedure to calculate $[CO_3^{2-}]_{EXP}$ and $[CO_3^{2-}]_{as}$ offset. From Gulf Stream to NADW or GNAIW, ALK and DIC are used as intermediate-step parameters to account for effects from freshwater inputs (step 1), biological respiration (step 2), and physical condition changes (step 3). Subscripts "surface" and "deep" represent surface and deep parameters, respectively. Superscripts "S" and "S-PO4" indicate values after S (step 1) and biological (step 2) corrections, respectively. C/PO_4 = soft-tissue Redfield stoichiometry, R = $C_{organic}$:C_{CaCO3} ratio.

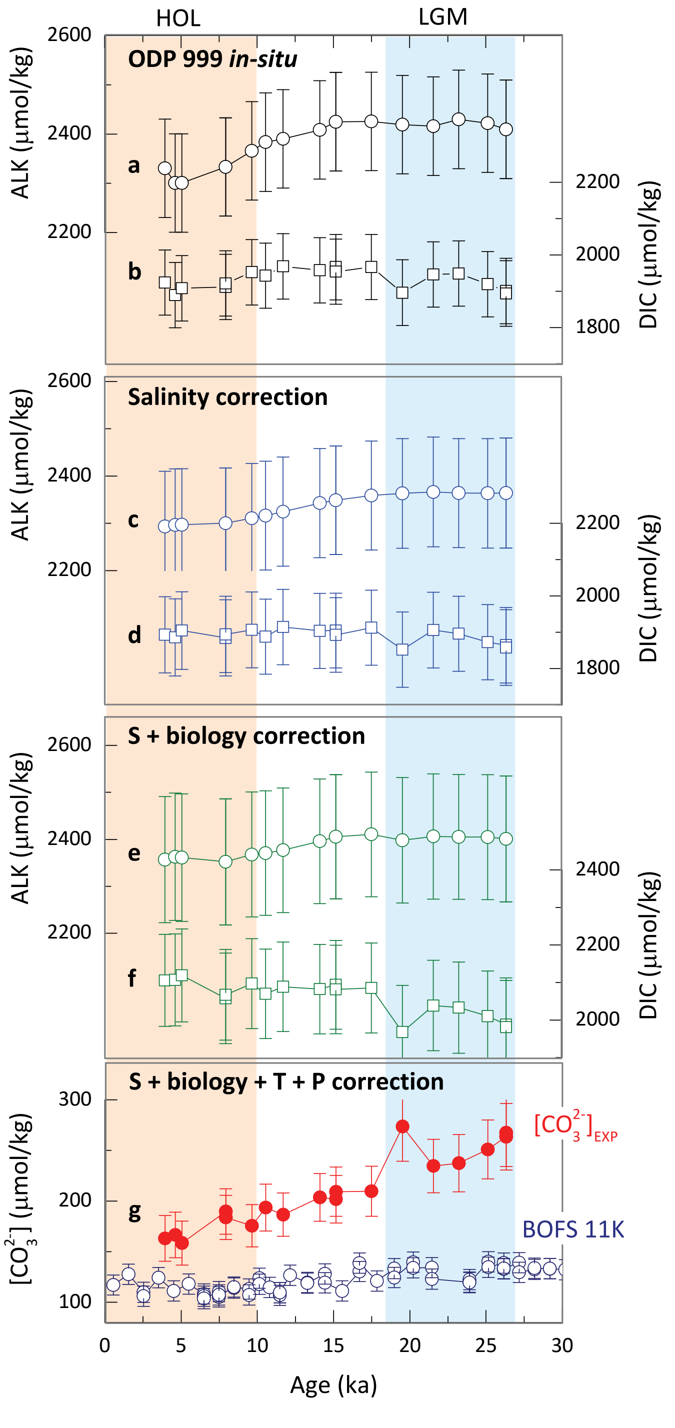

Supplementary Figure 10. Detailed $[CO_3^{2-}]_{EXP}$ calculations from ODP 999 to BOFS 11K. **a-b**, Surface water ALK and DIC at ODP 999. **c-d**, Salinity corrections, **e-f**, Salinity and biological corrections. **g**, $[CO_3^{2-}]_{EXP}$ compared to $[CO_3^{2-}]$ at BOFS 11K. Intermediate-step ALK and DIC values are shown. **Note** that variations in ALK and DIC are systematically related, given the constraint from pH reconstructions at ODP 999, allowing calculation of $[CO_3^{2-}]_{EXP}$ with acceptable precision. The mean fully propagated error for individual $[CO_3^{2-}]_{EXP}$ is ~±29 (HOL: ~25, LGM: ~33) µmol/kg, based on quadratic addition of $[CO_3^{2-}]$ uncertainties from individual errors from both surface ($T_{surface}$, $S_{surface}$, $ALK_{surface}$, and $\delta^{11}B_{borate}$) and deep water (T_{deep}, S_{deep}, and PO_{4deep}) variables. Error bars: ±2σ.

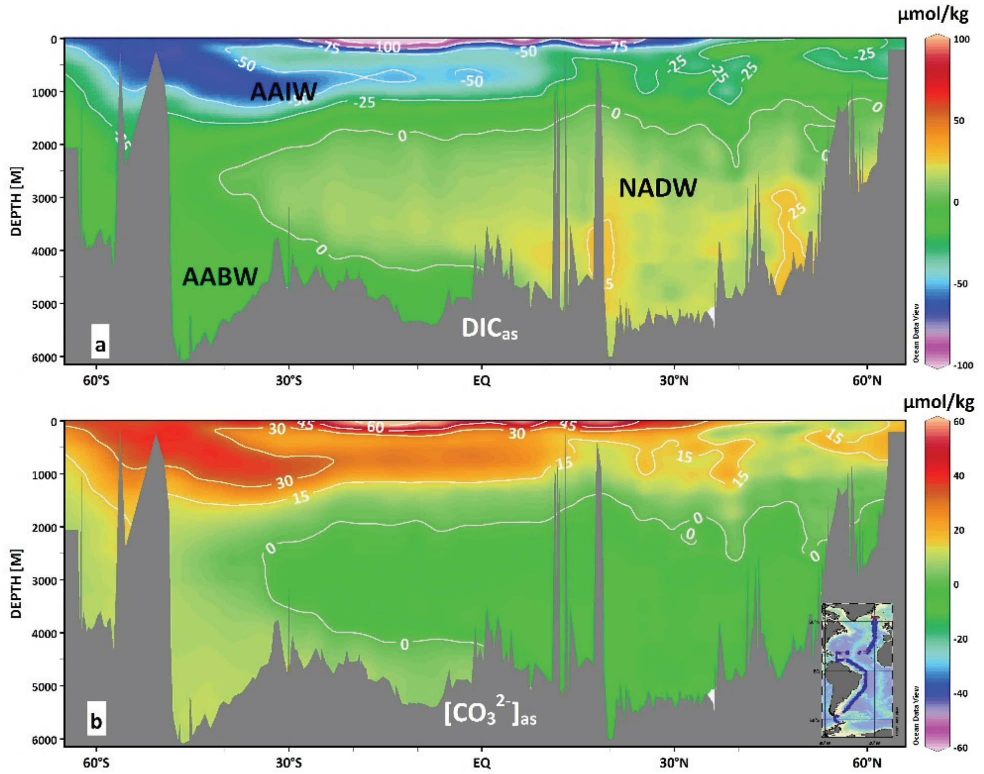

Supplementary Figure 11. Sensitivity test for C/PO$_4$; As Fig. 2, but using C/PO$_4$ = 106. Other parameters are the same as Fig. 2.

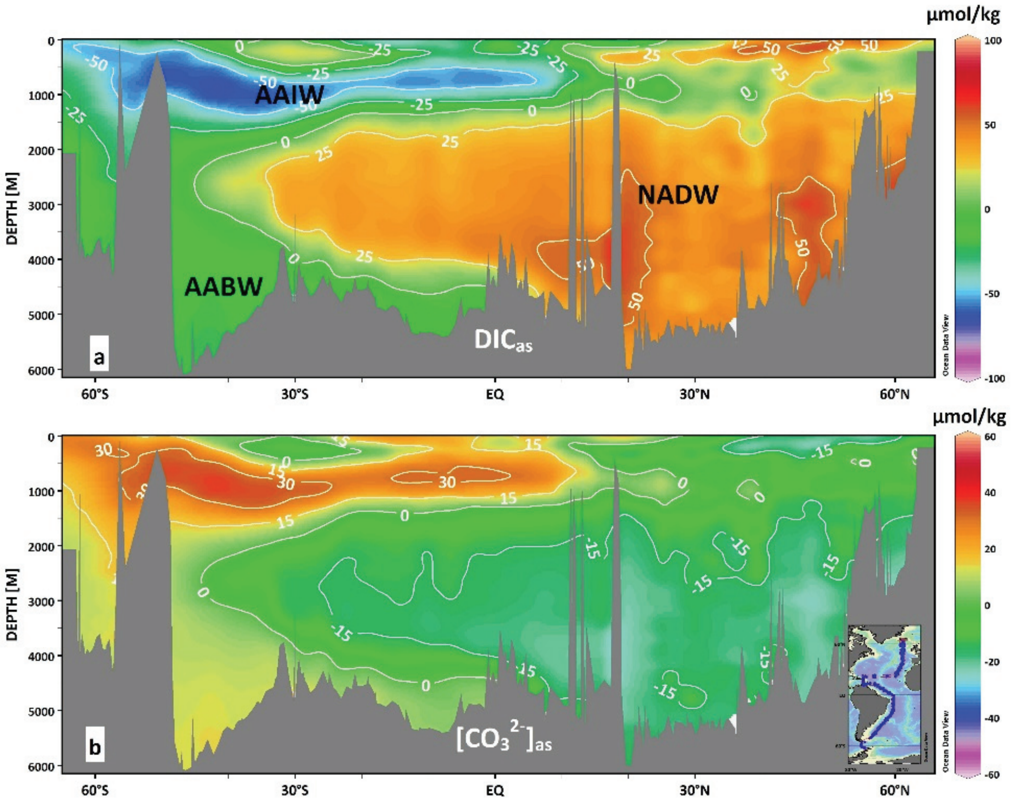

Supplementary Figure 12. Sensitivity test for C/PO4; As Fig. 2, but using C/PO4 = 140. Other parameters are the same as in Fig. 2.

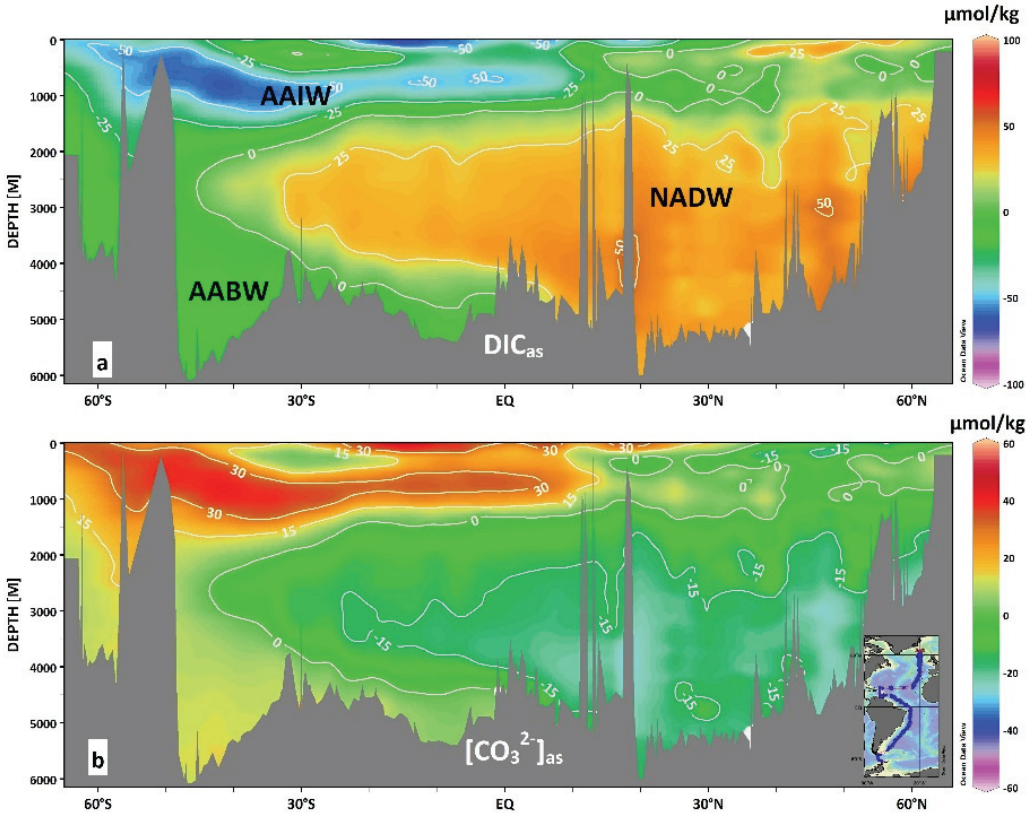

Supplementary Figure 13. Sensitivity test for C/PO4; As Fig. 2, but using a different reference composition. Instead of using global mean values (Fig. 2), we choose the following values (comparable to those in the mid-depth North Atlantic) for calculations using Equation (1): PO_4^{mo} = 1.3 µmol/kg, NO_3^{mo} =18 µmol/kg, ALK^{mo} = 2330 µmol/kg, $DIC_{constant}$ = 2135 µmol/kg, $[CO_3^{2-}]_{constant}$ = 130 µmol/kg, and C/PO4 = 127. The patterns are similar to those shown in Fig. 2.

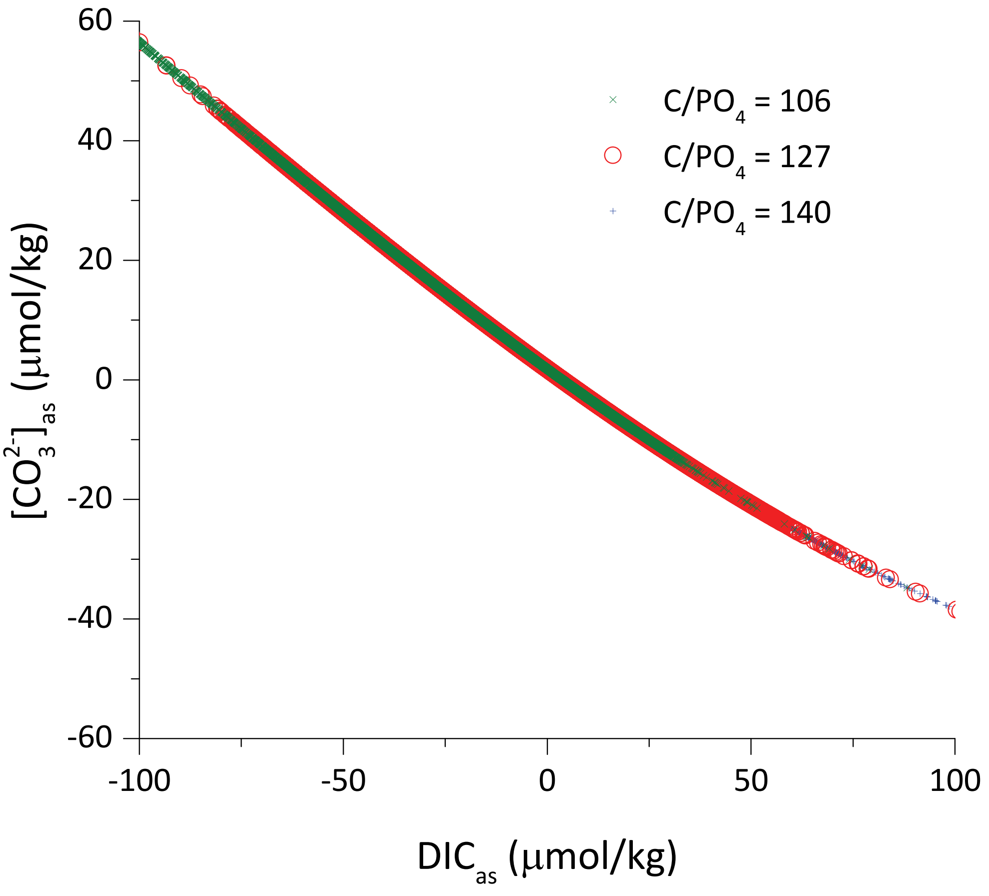

Supplementary Figure 14. Sensitivity test for C/PO$_4$; As Supplementary Figure 2, but for three C/PO$_4$ scenarios. Data are from 75°W to 15°E compiled by the GLODAP dataset (n = 14,078)[7]. The $[CO_3^{2-}]_{as}$/DIC_{as} slope is ~-0.5. The strong correlation demonstrates the use of $[CO_3^{2-}]_{as}$ to infer DIC_{as} changes.

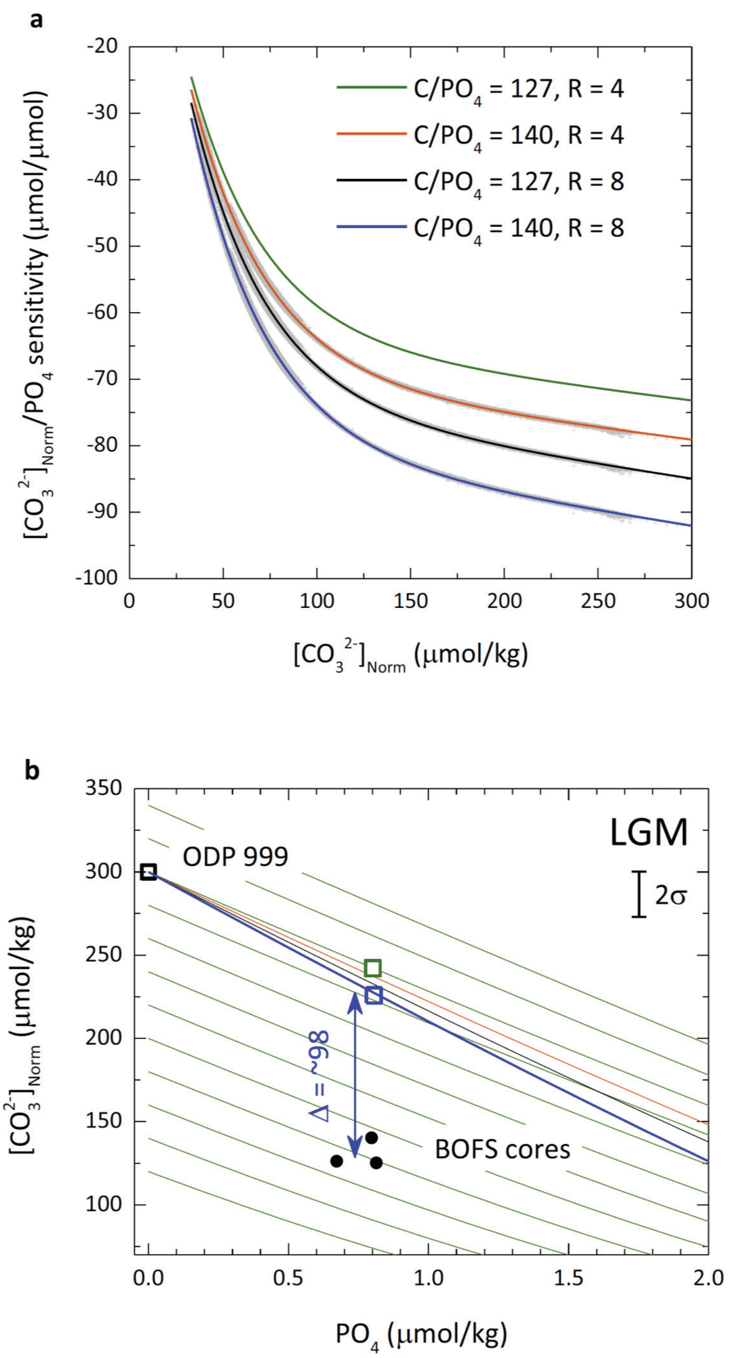

Supplementary Figure 15. C/PO4 and R effects on ODP 999- BOFS $[CO_3^{2-}]_{as}$ offsets. a, Redfield slopes for various C/PO4 and R values. Best fits for data are: C/PO4 =127, R = 8: $y = 54392*e^{-x/1280360} + 103*e^{-x/40} - 54465$; C/PO4 =140, R = 4: $y = 74394*e^{-x/2098450} + 95.2*e^{-x/40.2} - 74463$; C/PO4 =140, R = 8: $y = 90027*e^{-x/2030990} + 112.6*e^{-x/40.2} - 90106$. For all fits, $r^2 = 0.9988$ and n = 55,399 using GLODAP data (grey crosses)[7]. **b**, As Fig. 5b but only for LGM samples. ODP 999 $[CO_3^{2-}]_{Norm}$ at PO_4 = ~0.8 μmol/kg can be estimated using various biological slopes (color coding as in panel **a**). The blue curve represents the scenario where the minimum ODP 999- BOFS $[CO_3^{2-}]_{as}$ offset (~98) can be reached. Compared to the scenario in Fig. 5b, using C/PO4 =140 and R = 8 ***only*** for the LGM would reduce the ODP 999- BOFS $[CO_3^{2-}]_{as}$ offset by ~16 μmol/kg.

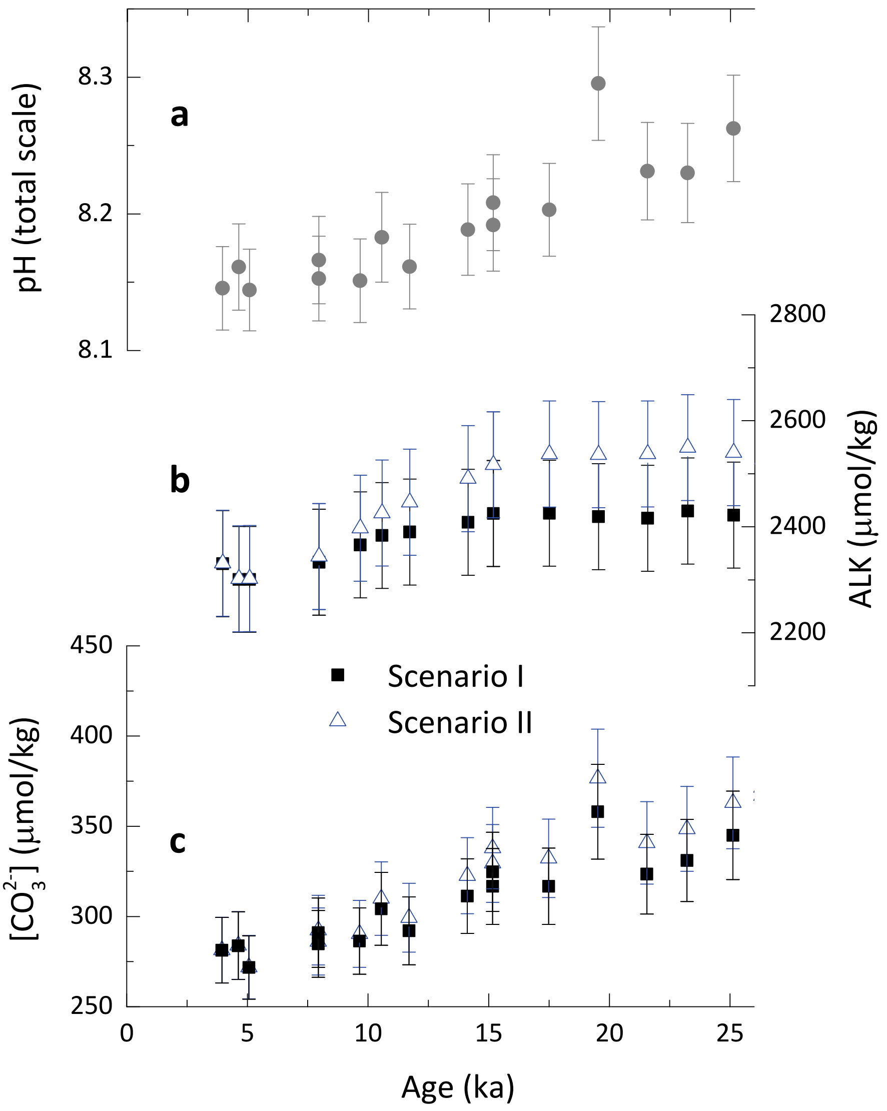

Supplementary Figure 16. ALK effect on surface-water [CO_3^{2-}] at ODP 999. a, pH derived from *G. ruber* δ^{11}B (refs [9,19]). **b**, ALK. **c**, [CO_3^{2-}]. Two scenarios are considered. Scenario I: past ALK changed only due to local salinity changes (black squares)[9] Scenario II: past ALK responded to both global and local ALK changes (blue triangles). Compared to the Holocene, the LGM deep ocean (>~1 km; mass = 1.04×10^{21} kg)[2] received ~500-700 PgC (ref. [20]), or 5.8×10^{16} molC, in total from the atmosphere (~200 PgC) and land biosphere (~300-500 PgC). Assuming an even deep ocean distribution, this carbon input would raise deep-sea DIC by ~40-56 μmol/kg. Given comparable global deep ocean [CO_3^{2-}] between the Holocene and LGM[21,22], the [CO_3^{2-}] effect from the glacial DIC gain ($\Delta DIC_{land+atmosphere}$ = ~40-56 μmol/kg) was buffered by $CaCO_3$ dissolution, a process that raises oceanic ALK (ΔALK_{CaCO3}) and DIC (ΔDIC_{CaCO3}) at a ratio of 2:1. Thus, we obtain: $\Delta ALK_{CaCO3} - \Delta DIC_{CaCO3} - \Delta DIC_{land+atmosphere} = \Delta ALK_{CaCO3} - \Delta ALK_{CaCO3}/2 - \Delta DIC_{land+atmosphere} = 0$; and $\Delta ALK = 2 \times \Delta DIC_{land+atmosphere}$ = ~80-112 μmol/kg. This ΔALK is independent of local ALK changes associated with the salinity change. Here, we assume that the global LGM ALK was 120 μmol/kg higher than during the Holocene, and declined in a pattern that mirrored sea level rise[23,24]. Error bars: ±2σ.

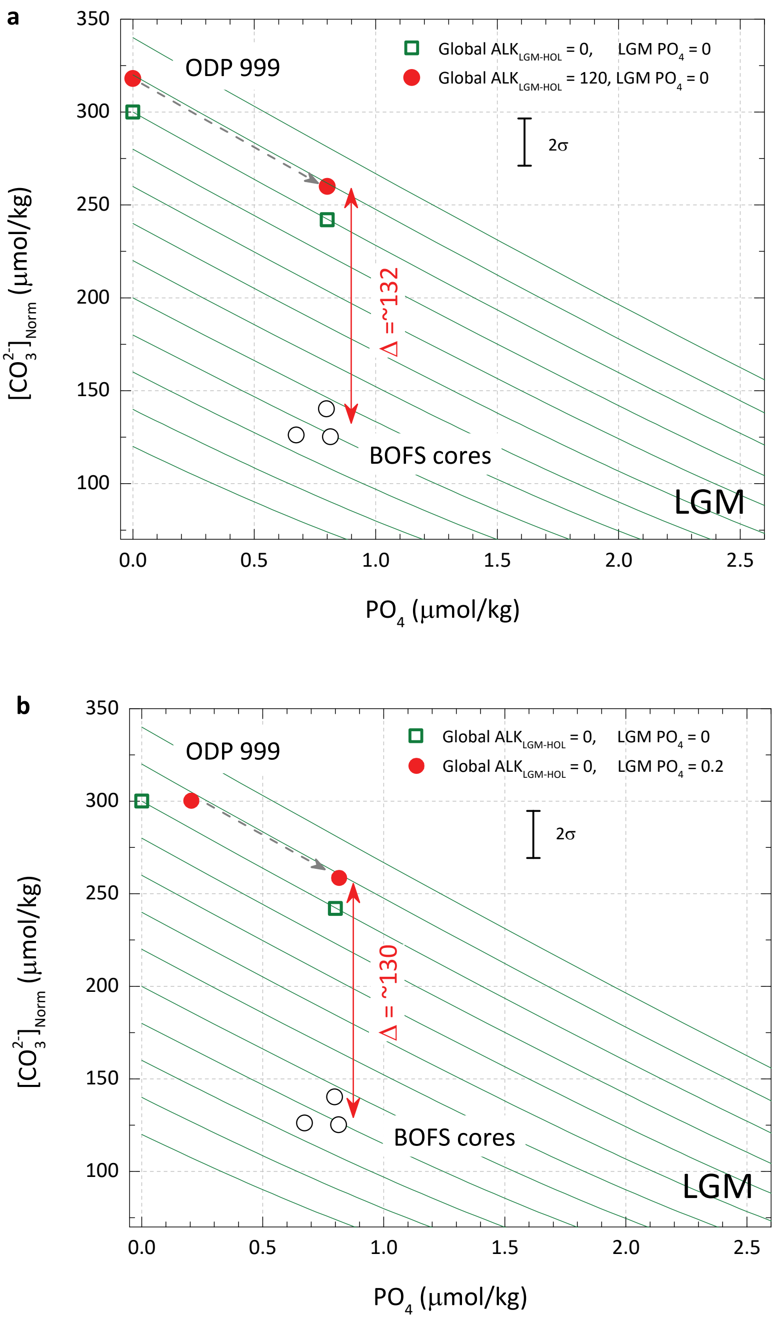

Supplementary Figure 17. ALK and surface-water PO_4 effects. a, Global ALK effect. **b**, Surface-water PO_4 effect. Green curves are biological trends using C/PO_4 = 127 and R = 4. Any increase in ALK and PO_4 at ODP 999 would raise the ODP 999- BOFS $[CO_3^{2-}]_{as}$ gradient during the LGM. This would suggest greater glacial North Atlantic carbon sequestration, strengthening our conclusions.

Supplementary Note 1

In addition to the pragmatic recipe presented in the main text, we describe a second approach to calculate $[CO_3^{2-}]_{as}$. The idea is the same as the pragmatic recipe, which is to compare water-mass $[CO_3^{2-}]$ values at the same T-S-P-PO_4 conditions. We use data from ODP Site 999 to predict the expected $[CO_3^{2-}]$ value, $[CO_3^{2-}]_{EXP}$, at the deep water site assuming no net air-sea CO_2 exchange and no mixing with other water masses during transformation of Gulf Stream waters into NADW/GNAIW. The approach involves three steps with frequent use of the CO_2sys.xls program[1] (Supplementary Figure 9). Conservative properties DIC and ALK are used as intermediate-step parameters to track seawater carbonate chemistry.

First, we correct the salinity effect on ALK and DIC due to fresh water inputs, using:

$$ALK_{surface}^{S} = ALK_{surface} \times S_{deep}/S_{surface} \quad (S1)$$

$$DIC_{surface}^{S} = DIC_{surface} \times S_{deep}/S_{surface} \quad (S2)$$

where $ALK_{surface}^{S}$ and $DIC_{surface}^{S}$ represent intermediate-step ALK and DIC values after the salinity correction, respectively.

Second, we consider influences on within-ocean carbon redistribution from respiration of biogenic matter on ALK and DIC. In the ocean, ALK and DIC are affected by both degradation of organic tissue and of $CaCO_3$. Following previous work[2-6], their effects can be estimated using C/PO_4 and R by:

$$ALK_{surface}^{S+PO4} = ALK_{surface}^{S} + \Delta[PO_4]_{deep\text{-}surface} \times C/PO_4 \div R \times 2 - \Delta[PO_4]_{deep\text{-}surface} \times N/PO_4, \quad \text{and} \quad (S3)$$

$$DIC_{surface}^{S+PO4} = DIC_{surface}^{S} + \Delta[PO_4]_{deep\text{-}surface} \times C/PO_4 + \Delta[PO_4]_{deep\text{-}surface} \times C/PO_4 \div R \quad (S4)$$

where $ALK_{surface}^{S+PO4}$ and $DIC_{surface}^{S+PO4}$ represent intermediate-step ALK and DIC values after salinity and biological corrections, respectively; $\Delta[PO_4]_{deep\text{-}surface}$ represents the PO_4 difference between deep and surface waters; C/PO_4, N/PO_4, and R stand for molar ratios of C to PO_4,

nitrogen to PO_4, and $C_{organic}$ to $CaCO_3$, respectively; terms $\Delta[PO_4]_{deep-surface} \times C/PO_4$ and $\Delta[PO_4]_{deep-surface} \times C/PO_4 \div R$ correct for DIC changes due to soft-tissue respiration and $CaCO_3$ dissolution, respectively; and terms $\Delta[PO_4]_{deep-surface} \times C/PO_4 \div R \times 2$ and - $\Delta[PO_4]_{deep-surface} \times N/PO_4$ correct for ALK changes created by $CaCO_3$ dissolution and soft-tissue respiration, respectively. Because $[PO_4]_{surface}$ is assumed to be zero, $\Delta[PO_4]_{deep-surface} = [PO_4]_{deep}$. Throughout, it is assumed that $[PO_4]_s = 0$ and $N/PO_4 = 16$. We use a reference Redfield C/PO_4 ratio of 127 and R of 4 for calculations shown in Supplementary Figure 10.

Third, $ALK_{surface}^{S+PO4}$ and $DIC_{surface}^{S+PO4}$, together with T_{deep} and S_{deep}, are used to calculate $[CO_3^{2-}]_{EXP}$ values at the deep ocean site of interest (e.g., P = 2004 dbar at BOFS 11K), using the CO_2sys program[1].

$[CO_3^{2-}]_{EXP}$ represents the expected $[CO_3^{2-}]$ of ODP 999 surface waters after incorporating biological and physical influences from ODP 999 to BOFS sites, but differs from reconstructed $[CO_3^{2-}]$ at BOFS sites because it is calculated assuming no air-sea CO_2 exchange during transformation of the Gulf Stream into NADW/GNAIW (Supplementary Figure 9). Therefore, the difference between $[CO_3^{2-}]_{EXP}$ and reconstructed deep-water $[CO_3^{2-}]$ reflects the ODP 999−BOFS $[CO_3^{2-}]_{as}$ contrast. For BOFS 11K, ODP 999−BOFS $[CO_3^{2-}]_{as}$ contrasts are ~59 μmol/kg and ~122 μmol/kg for the Holocene and LGM, respectively (Supplementary Figure 10). This suggests an increase in $[CO_3^{2-}]_{as}$ gradient of ~63 μmol/kg during the LGM, similar to the results (~58 μmol/kg) based on the pragmatic recipe (Fig. 5).

Supplementary References

1. Pelletier G, Lewis E, Wallace D. *A calculator for the CO_2 system in seawater for Microsoft Excel/VBA*, 1.0 edn. Washington State Department of Ecology, Olympia, WA: Brookhaven National Laboratory, Upton, NY, 2005.

2. Sarmiento JL, Gruber N. *Ocean Biogeochemical Dynamics*. Princeton University Press: Princeton, 2006.

3. Sigman DM, McCorkle DC, Martin WR. The calcite lysocline as a constraint on glacial/interglacial low-latitude production changes. *Glob Biogeochem Cycle* 1998, **12**(3)**:** 409-427.

4. Archer D, Maier-Reimer E. Effect of deep-sea sedimentary calcite preservation on atmospheric CO_2 concentration. *Nature* 1994, **367**(6460)**:** 260-263.

5. Broecker WS, Peng TH. The role of $CaCO_3$ compensation in the glacial to interglacial atmospheric CO_2 change. *Glob Biogeochem Cycle* 1987, **1**(1)**:** 15-29.

6. Boyle E. The role of vertical chemical fractionation in controlling late Quaternary atmospheric carbon dioxide. *J Geophys Res* 1988, **93**(C12)**:** 15701-15714.

7. Key RM, Kozyr A, Sabine CL, Lee K, Wanninkhof R, Bullister JL*, et al.* A global ocean carbon climatology: Results from Global Data Analysis Project (GLODAP). *Glob Biogeochem Cycle* 2004, **18**(4)**:** doi: 10.1029/2004GB002247.

8. Schlitzer R. Ocean Data View. 2006, **http://odv.awi-bremerhaven.de**.

9. Henehan MJ, Rae J, Foster GL, Erez J, Prentice KC, Kucera M*, et al.* Calibration of the boron isotope proxy in the planktonic foraminifera *Globigerinoides ruber* for use in palaeo-CO_2 reconstruction. *Earth Planet Sci Lett* 2013, **364:** 111-122.

10. Schmidt MW, Spero HJ, Lea DW. Links between salinity variation in the Caribbean and North Atlantic thermohaline circulation. *Nature* 2004, **428**(6979)**:** 160-163.

11. Foster GL, Sexton PF. Enhanced carbon dioxide outgassing from the eastern equatorial Atlantic during the last glacial. *Geology* 2014, **42**(11)**:** 1003-1006.

12. Schlitzer R, Anderson RF, Dodas EM, Lohan M, Geibert W, Tagliabue A*, et al.* The GEOTRACES Intermediate Data Product 2017. *Chem Geol* 2018, **493:** 210-223.

13. Elderfield H, Rickaby REM. Oceanic Cd/P ratio and nutrient utilization in the glacial Southern Ocean. *Nature* 2000, **405**(6784)**:** 305-310.

14. Abe K. Preformed Cd and PO4 and the relationship between the two elements in the northwestern Pacific and the Okhotsk Sea. *Mar Chem* 2002, **79**(1)**:** 27-36.

15. Abe K. Cd in the western equatorial Pacific. *Mar Chem* 2001, **74**(2-3)**:** 197-211.

16. Abouchami W, Galer SJG, de Baar HJW, Alderkamp AC, Middag R, Laan P*, et al.* Modulation of the Southern Ocean cadmium isotope signature by ocean circulation and primary productivity. *Earth Planet Sci Lett* 2011, **305**(1-2)**:** 83-91.

17. Boyle EA. Cadmium: Chemical tracer of deepwater paleoceanography. *Paleoceanogr* 1988, **3:** 471-489.

18. Came RE, Oppo DW, Curry WB, Lynch-Stieglitz J. Deglacial variability in the surface return flow of the Atlantic meridional overturning circulation. *Paleoceanogr* 2008, **23**(1)**:** Artn Pa1217.

19. Foster GL. Seawater pH, pCO_2 and [CO_3^{2-}] variations in the Caribbean Sea over the last 130 kyr; a boron isotope and B/Ca study of planktic foraminifera. *Earth Planet Sci Lett* 2008, **271**(1-4)**:** 254-266. doi: 210.1016/j.epsl.2008.1004.1015.

20. Sigman DM, Boyle EA. Glacial/interglacial variations in atmospheric carbon dioxide. *Nature* 2000, **407**(6806)**:** 859-869.

21. Yu J, Anderson RF, Jin ZD, Rae J, Opdyke BN, Eggins S. Responses of the deep ocean carbonate system to carbon reorganization during the Last Glacial–interglacial cycle. *Quat Sci Rev* 2013, **76:** 39-52.

22. Anderson DM, Archer D. Glacial-interglacial stability of ocean pH inferred from foraminifer dissolution rates. *Nature* 2002, **416**(6876)**:** 70-73.

23. Lambeck K, Rouby H, Purcell A, Sun YY, Sambridge M. Sea level and global ice volumes from the Last Glacial Maximum to the Holocene. *P Natl Acad Sci USA* 2014, **111**(43)**:** 15296-15303.

24. Stanford JD, Rohling EJ, Hunter SE, Roberts AP, Rasmussen SO, Bard E*, et al.* Timing of meltwater pulse 1a and climate responses to meltwater injections. *Paleoceanogr* 2006, **21**(4)**:** doi: 10.1029/2006pa001340.
