# Supplementary material for: More efficient North Atlantic carbon pump during the Last Glacial Maximum
Source: Nat Commun. 2019 May 15;10:2170. doi: 10.1038/s41467-019-10028-z (PMC6520411; doi:10.1038/s41467-019-10028-z)
Supplement: Supplementary file 3 — Description of Additional Supplementary Files [file 41467_2019_10028_MOESM3_ESM.pdf]

Description of Additional Supplementary Files

File name: Supplementary Data 1

Description: Surface water results at ODP Site 999

File name: Supplementary Data 2

Description: Surface water [CO32-] for sites from subtropical western Atlantic

File name: Supplementary Data 3

Description: Deep water results in core BOFS 17K

File name: Supplementary Data 4

Description: Deep water results in core BOFS 14K

File name: Supplementary Data 5

Description: Deep water results in core BOFS 11K

File name: Supplementary Data 6

Description: Average results for the Holocene and LGM

File name: Supplementary Data 7

Description: Redfield slopes reference curves

File name: Supplementary Data 8

Description: Template to calculate [CO32-]_{Norm} and [CO32-]_{Norm, PO4=x}

File name: Supplementary Data 9

Description: Data references used in Supplementary Data
